# Electrochemical Biosensors Based on Nanomaterials for Early Detection of Alzheimer’s Disease

**DOI:** 10.3390/s20174748

**Published:** 2020-08-22

**Authors:** Celia Toyos-Rodríguez, Francisco Javier García-Alonso, Alfredo de la Escosura-Muñiz

**Affiliations:** 1 NanoBioAnalysis Group-Department of Physical and Analytical Chemistry, University of Oviedo, Julián Clavería 8, 33006 Oviedo, Spain; toyoscelia@uniovi.es; 2 Biotechnology Institute of Asturias, University of Oviedo, Santiago Gascon Building, 33006 Oviedo, Spain; fjga@uniovi.es; 3 NanoBioAnalysis Group-Department of Organic and Inorganic Chemistry, University of Oviedo, Julián Clavería 8, 33006 Oviedo, Spain

**Keywords:** Alzheimer’s disease, neurodegenerative diseases, nanoparticles, labels, electroactivity, electrocatalysis, electrochemistry, immunosensors, biosensors, nanomaterials

## Abstract

Alzheimer’s disease (AD) is an untreatable neurodegenerative disease that initially manifests as difficulty to remember recent events and gradually progresses to cognitive impairment. The incidence of AD is growing yearly as life expectancy increases, thus early detection is essential to ensure a better quality of life for diagnosed patients. To reach that purpose, electrochemical biosensing has emerged as a cost-effective alternative to traditional diagnostic techniques, due to its high sensitivity and selectivity. Of special relevance is the incorporation of nanomaterials in biosensors, as they contribute to enhance electron transfer while promoting the immobilization of biological recognition elements. Moreover, nanomaterials have also been employed as labels, due to their unique electroactive and electrocatalytic properties. The aim of this review is to add value in the advances achieved in the detection of AD biomarkers, the strategies followed for the incorporation of nanomaterials and its effect in biosensors performance.

## 1. Introduction

Alzheimer’s disease (AD) is a debilitating neurodegenerative disorder and the current main cause of dementia [1]. This untreatable disease affected more than 50 million people in 2019 and this number is estimated to increase to 152 million by 2050 according to Alzheimer’s disease international (ADI) and the World health organization (WHO) [2]. Even though AD affects mainly older people, it is considered a multifactorial disease that appears before 65 years in 4% of cases. Commonly, AD starts with an initial phase, known as mild cognitive impairment (MCI), characterized by unclear episodes of memory and non-memory related impairments, that evolves to symptoms of dementia, implying deterioration of cognitive functions, memory loss, inability to perform daily tasks or time and space disorientation [3,4]. Cognitive decline associated with AD has a significant impact not only in patients, but also in families and careers, affecting them both emotionally and economically. In 2015, the Global Economic Impact of AD was of 818 billion US dollars, nearly 1.1% of the Gross Domestic Product (GDP) and it is expected to grow to two trillion US dollars by 2030 [5].

Due to its socio-economic effect, the Group of eight (G8) inter-governmental political forum stated in 2013 that discovering a therapy before 2025 was a global priority [1]. Unfortunately, there is still no treatment for AD, although recent research has been focused on disease-modifying drugs for early stages of the disease owing to its increased efficiency [1,6], what highlights the importance of an early detection. The diagnose of AD can be performed by conducting cognitive tests and by imaging techniques, mainly magnetic resonance imaging (MRI), positron emission tomography (PET) and near infrared (NIR), used to detect abnormalities in patient brains [7]. The analysis of cerebrospinal fluid (CSF) and blood plasma biomarkers by immunohistochemistry and enzyme linked immunosorbent assay (ELISA) have also been used [8]. These techniques are time-consuming, expensive and invasive and do not constitute a generalize method for an early detection of AD. Due to that, there is a still a need to develop easy-to-use, low-cost, sensitive methods to facilitate the detection of AD and that require less amount of sample to minimize the extraction procedures performed on patients, as it is the case of electrochemical biosensors. In this review, electrochemical biosensors for the detection of AD are revised, according to the biomarkers detected and focusing on the use of nanomaterials in these analytical devices to improve biosensors performance.

## 2. Biomarkers for Alzheimer’s Disease

AD is a multifactorial disease that evolves progressively until the first symptoms of dementia appear with variable clinical among patients. This fluidity difficulties detection only by cognitive tests, favoring misdiagnosis and delaying medication administration [8]. Due to that, biomarkers have gained importance for an early diagnosis of AD.

Even though molecular bases of AD are not well established, amyloid beta (Aβ) peptides plaques and intracellular neurofibrillary tangles (NFTs) of hyperphosphorylated microtubule-associated protein tau (MAPT) are the most accepted pathological hallmarks of the disease.

Aβ plaques are composed by aggregated Aβ peptides, generated by the proteolysis of amyloid precursor protein (APP), a transmembrane type I glycoprotein that is present in almost all tissues and whose physiological function is still unknown [9]. The cleavage of APP occurs by two different pathways: amyloidogenic, which is the causative of amyloidogenic diseases, including AD, and non-amyloidogenic [10]. In the amyloidogenic pathway, APP is cleaved by β-secretase (identified as BACE1) and γ-secretase (composed of four proteins including presenilin protein) on the N- and C-terminal ends, generating peptides of 39–43 amino acids [11,12], Aβ(1-40) and Aβ(1-42) being predominant. However, this cleavage occurs only in 10–20% of cases, as the predominant cleavage pathway (non-amyloidogenic) is performed by α-secretase between 16 and 17 residues [13], generating non-amyloidogenic peptides. Any imbalance in the generation or clearance of amyloidogenic Aβ peptides leads to Aβ accumulation and constitutes a risk factor for AD development. Mutations in the APP gene and in presenilin gene have been related with an increased Aβ production and are associated with familial AD, the main cause of early onset dementia [14,15].

After Aβ peptide generation, Aβ monomers tend to form aggregates first in the form of oligomers (Aβo), protofibrils and then fibrils (Aβf) that accumulate extracellularly in neuron cell surfaces forming senile plaques. According to the “amyloid-cascade hypothesis” postulated by Hardy and Higgins in 1992 [16] the accumulation of Aβ forming plaques was the main cause of neurotoxicity and dementia. However, later studies have reported that some normal cognitive patients also possessed senile plaques [17,18], suggesting that there are other components involved in the onset of AD. Free soluble Aβo have been detected in CSF samples of AD patients [19] and have been found to produce cognitive impairments in patients without plaques [20]. The toxicity of Aβo has been related with the capacity of these molecules to cross the lipid bilayer of neuronal cells, inducing the depolarization of neurons’ membranes by an allowable influx of Ca^2+^ ions leading to synaptic failure [21,22,23].

Another important hallmark is NFTs of MAPT, that are generated by the hyperphosphorylation of tau protein [24]. Tau protein is the main microtubule-associated protein of neurons involved in the assembly of tubulin. Normal phosphorylation of tau protein regulates axonal growth, transport and neuronal polarity. In several neurodegenerative diseases, tau is hyperphosphorylated and tends to aggregate forming pair helical filaments (PHFs), that form intracellular NFTs, neuropil threads and dystrophic neurons extracellularly accumulated near Aβ plaques [24,25,26,27,28].

Aβ and fibrillary tangles are not the only biomarkers currently object of study. ApoE4 has been postulated for an early detection of AD as it is considered the major genetic risk factor for AD. ApoE is a glycoprotein that acts as a ligand in mediated endocytosis of lipoprotein particles and it is mainly expressed in the brain in astrocytes and microglia [29]. Human ApoE has three isoforms (ApoE2, ApoE3 and ApoE4) that differ in the presence of arginine or cysteine amino acids at positions 112 and 158 [30], being E4 directly associated with AD, especially with an earlier age onset [31]. ApoE4 in AD is considered to act as a binding protein for Aβ, inducing the formation of pathological β-sheets [32]. Aβ, hyperphosphorylated Tau and ApoE4 have all been found in CSF samples of AD patients [33] constituting reliable biomarkers of the disease with a sensitivity of 85–90% in early onset AD.

AD pathogenesis has also been related with novel biomarkers as p53 protein, since Lanni et al. [34] observed in 2007 that unfolded p53 peptide is highly expressed in fibroblast of non-AD patients in the presence of nanomolar concentrations of Aβ peptide, suggesting that the presence of low and non-toxic levels of this biomarker could induce cell changes, including the formation of an abnormal tertiary conformation of p53 which must appear before the start of amyloidogenic cascade. In its normal conformation, p53 is considered as the “guardian of the genome” as it is a multifunctional protein with antioxidant activity involved in tumoral growth suppression [35], while its unfolded conformation has been studied owing to its pathogenicity [36,37,38].

Moreover, neurotransmitters have also been related with the development of neurodegenerative diseases, including AD, as they may interact with Aβo [39]. Acetylcholine (Ach), dopamine (DA) and norepinephrine (NE) have been studied as potential biomarkers for the evaluation of neurodegenerative diseases, not only for an early detection but also to evaluate potential drug candidates [40,41,42].

Oxidative stress has also been proposed as hallmark since inflammation, Aβ accumulation or hyperphosphorylated tau could induce the process [43]. When this happens, an increase in oxidative stress biomarkers (H_2_O_2_ or superoxide dismutase (SOD) among others) in blood is reported [44].

All the above mentioned biomarkers have been summarized in Table 1, as well as less extended AD biomarkers as microRNAs (miRNAs), BACE1 [45] or α-1-antitripsine [46], a protein related with Aβ peptide fibril formation [47], that have also been investigated by electrochemical means.

With regard to the samples measured, most of the biomarkers studied so far are present in CSF and blood samples, as has been stated above, but there is an increasing tendency to use salivary samples, both to detect conventional biomarkers and others like lactoferrin specific for this type of samples [48]. However, it is still needed further investigation before their application in biosensing.

Although the identification of AD biomarkers is a complex and extensive field, diagnosis criteria vary as further as investigations are performed. What is clear is that as important as knowing the appropriate biomarkers is being able to identify them, for what electrochemical biosensors emerge as outstanding tools.

## 3. Electrochemical Biosensors: The Role of Nanomaterials

### 3.1. General Overview

Electrochemistry is defined as the branch of chemistry implicated in the interrelation between electrical and chemical effects [53], which includes the study of the production of electricity by chemical reactions and the chemical changes generated by the passage of an electrical current. Both these phenomena are used in biosensors development. On the other hand, a biosensor is defined as a device that converts biological information into measurable analytical signals [54]. Biosensors are composed of a molecular recognition element (receptor) and a physicochemical transducer, that in the case of electrochemical biosensors is an electronic conducting, semi-conducting or ionic conducting material, that allows measuring analytical samples by different techniques including potentiometry, amperometry, conductometry and field-effect.

With regard to the electrochemical recognition process employed, biosensors may be classified in two groups, those that use a biocatalytic recognition element, namely enzymes, cells and tissues, and those based on bioaffinity reactions, which includes immunosensors (using antigen-antibody interactions), genosensors (employing nucleic acids as recognition element) and aptamer-based biosensors (sequence of synthetic oligonucleotides as biological recognition element) [54,55,56]. Electrochemical immunosensors have been widely studied as the high affinity reached by antigen-antibody interactions improves specificity and allows the detection of trace amounts of biomarkers. Deoxyribonucleic acid (DNA) and ribonucleic acid (RNA) sensors, in contrast, are characterized by having a sequence of oligonucleotides (probe) as biological recognition element [55,57]. The principle of these sensors is based on the specific hybridization between the oligonucleotides immobilized in the transducer and the complementary analyte in such a way that they could be applied for the detection of polymorphisms in the DNA and RNA associated with genetic-related diseases. Compared to traditional techniques for the detection of DNA and RNA sequences that include polymerase chain reaction (PCR) [58] or fluorescence in situ hybridization (FISH) [59], biosensors are simpler, less time-consuming and more sensible. Due to that, they have gained attention for the detection of circulating biomarkers, including those related to AD disease.

Among bioaffinity sensors, aptamer-based ones have gained special interest as they have an enhanced efficiency in contrast to antibodies, as they are more stable, easier to bioconjugate and less toxic [60,61]. However, selection process of aptamers is slightly more tedious as selective evolution of ligands by exponential enrichment (SELEX) has to be performed, so that antibodies are still the most chosen option.

An appropriate immobilization of biological recognition elements on the transducer is a key aspect in bioaffinity sensors, as the performance is dependent on the coating obtained. With the aim of improving this aspect, nanomaterials have been extensively used.

Overall, due to their reduced size, fast operation, sensitivity and low cost, electrochemical biosensors are point-of-care (POC) devices suitable for replacing currently used laboratory analysis [62]. It is also worthy to highlight the low sample volume required by most of the electrochemical biosensing configurations (typically around 10 µL). This is of special relevance for the analysis of samples like blood and CSF which require invasive extraction.

### 3.2. The Role of Nanomaterials

Although electrochemical biosensors have gained importance as POC devices over the past few years, it is still necessary to enhance biosensors performance in order to lower detection limits. For that purpose, nanomaterials have been proved to be useful [63] both as electrode modifiers and as labels, due to their high electron transfer, biocompatibility and electrocatalytic activity, among other properties.

In the last decades, significant progress has been made in the synthesis of nanomaterials with tailored characteristics (size, shape or surface charge) and on their functionalization and combination with other nanomaterials, polymers or biomolecules [64].

As electrode modifiers, nanomaterials have mainly been incorporated following two objectives: improving the electronic transference and anchoring biological recognition elements.

The immobilization of biomolecules in nanomaterials has a huge impact on the reproducibility, robustness of the assay and increased time stability, owing to its strong absorption capacity and its biocompatibility compared to bulk materials.

Coating the transducer with nanomaterials functionalized with biomolecules allows an appropriate orientation of the biological recognition elements, which is of key relevance for ensuring that the binding sites are accessible and that the redox-active sites in the biomolecule are in contact with the transducer [65,66,67,68].

**Figure 1:**
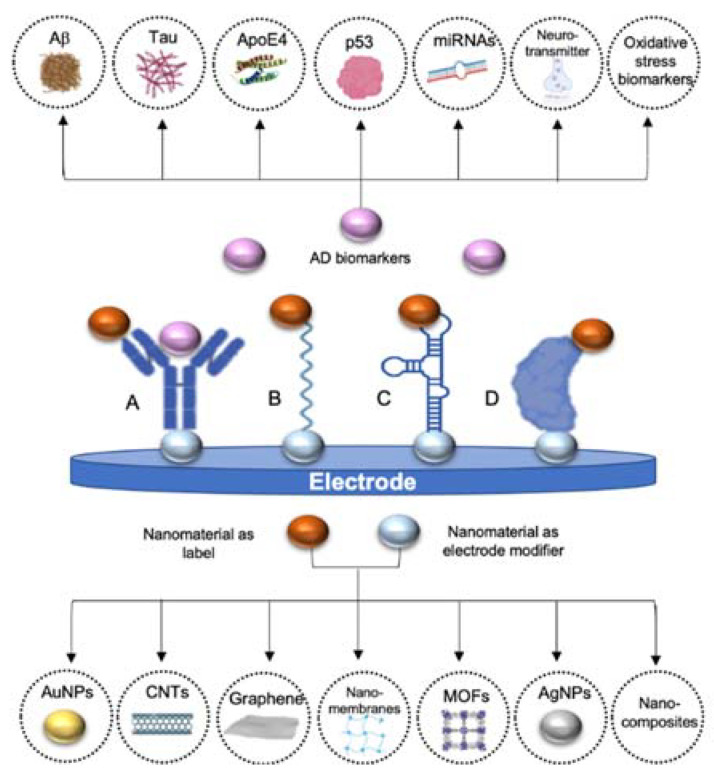
Schematic representation of the main AD biomarkers and the typical nanomaterials used in electrochemical biosensors for AD detection (gold nanoparticles (AuNPs), carbon nanotubes (CNTs), graphene, nanomembranes, metal-organic frameworks, silver nanoparticles (AgNPs) and nanocomposites). A. Using antibodies as recognition element, B. using oligonucleotides as recognition element, C. using aptamers as recognition element and D. using affinity proteins as recognition elements. In all cases nanomaterials may be used both to modify the electrode and as label, not being necessary the use of both nanomaterials at the same time in all the detection strategies.

Additionally, nanomaterials have also been used as labels, including metal nanoparticles, carbon-based nanomaterials or electroactive nanovesicles, due to their electrochemical and electrocatalytic properties [69,70]. Even though enzyme-based labels are most common, their low thermal stability and non-conductivity have favored its substitution by nanomaterials. In comparison, nanomaterials have higher stability, signal amplification capacity and sensitive electrochemical response than traditional enzymes or metal ion probes [71,72]. Their particular characteristics compared to those of the bulk materials have raised them as fundamental parts of electrochemical biosensors as they are ideal tools for improving sensitivity, time stability and lower limits of detection (LOD). In Figure 1, nanomaterials mostly used in AD biosensors, both as electrode modifiers and as labels, have been represented, thereby AD biomarkers detected through electrochemical biosensors. Principal strategies followed for the detection of AD biomarkers are also outlined, including the use of antibodies, DNA probes, aptamers and proteins as recognition elements.

## 4. Electrochemical Biosensors for Alzheimer’s Disease Diagnostics

The increasing incidence of AD has turned it into the most studied neurodegenerative disease. The presence of AD biomarkers in blood and CSF has promoted the development of electrochemical biosensors for their detection, as they allow the obtention of quantitative results in five minutes to two hours [48]. However, reaching the low cut-off values of the main AD biomarkers in human samples and improving selectivity and time-stability of biosensors are the heading challenges here [73]. For that purpose, nanomaterials have been introduced in biosensors either as labels or electrode modifiers.

As it can be deduced from the works revised in this review, peptides are, by far, the most widely detected biomarkers followed by proteins, as Aβ peptide is, till now, the mainly accepted biomarker. With regard to biological recognition elements, antibodies are predominant even though the use of aptamers is remarkable as well as the combination of these two elements in sandwich type biosensors. From nanomaterials, AuNPs are, without no doubt, the most frequently used materials, both as electrode modifiers and as labels, followed by carbon-based nanomaterials. It is worth remarking that the use of combinations of different nanomaterials, known as nanocomposites, is increasing as in the majority of cases they present a synergistic effect.

Below are described the most representative electrochemical biosensors for the detection of AD biomarkers in which nanomaterials are involved, classified according to the analyte measured: DNA or RNA, peptides, proteins, neurotransmitters and oxidative stress biomarkers, and attending to the nanomaterials used and the contribution they made to biosensors performance.

### 4.1. DNA and RNA Biomarkers

DNA is the carrier of genetic information, that differs in every single organism making it unique. During cells life cycle many mutations can occur, having a severe impact in humans health in a hereditary way [74]. Since the discovery of DNA double helix structure [75], the identification and sequencing of this genetic information have been of top interest to both understand and diagnose genetic diseases. In the case of AD, ApoE4 gene is the main genetic biomarker, as it has been related to late-onset familial AD [76]. Moreover, not only DNA sequences have been used as nucleic acid biomarkers. MicroRNAs (miRNAs) have been a hot topic since their identification in plasma and serum human samples in 2008 [77]. MiRNAs are single-stranded, non-coding short oligonucleotides (~22 nucleotides) that regulate the translational expression of their complementary target messenger RNA (mRNA) by specifically binding to them on the 3′ untranslated regions (UTRs) [78]. An aberrant expression of miRNAs can lead to the appearance of various diseases, including AD, in which it has been hypothesized that the downregulation of these sequences could be related with a pathological regulation of APP and presenilins 1 and 2 genes [51,52]. Owing to the great potential of miRNAs, the development of electrochemical biosensors for their detection have increased in recent years [79].

#### 4.1.1. Nanomaterials as Electrode Modifiers

Graphene and its derivatives have been the most extensively used nanomaterials to modify the electrode in AD immunosensors. Graphene is a two-dimensional (2D) sheet of carbon atoms with sp2 hybridization which has gained importance since its isolation in 2004 due to its mechanical strength and thermal and electrical conductivity [80]. Since its discovery, it has been investigated for a wide range of applications including biosensors development.

**Figure 2:**
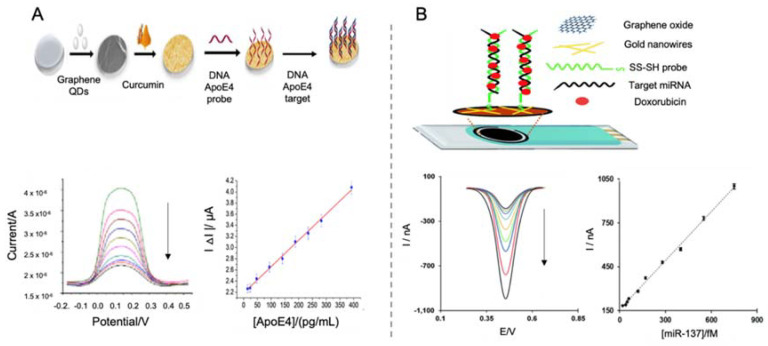
Electrochemical biosensors for the detection of DNA and RNA biomarkers of AD. (**A**). Schematic representation of a ITO electrode modified with graphene QDs, electropolymerized curcumin as reporter signal and DNA ApoE4 probe for the detection of ApoE4 gene using a DNA probe as recognition element (top) and differential pulse voltammetric (DPV) curves of the detected ApoE4 at different concentrations, representing an increase in the resistance with higher concentrations of ApoE4 and the corresponding calibration curve (bottom). Reprinted from [83]. Copyright (2018) with permission from Elsevier. (**B**). Schematic representation of miRNA biosensor based on the modification of a SPCE using GO, gold nanowires, thiolated RNA probe as recognition element and doxorubicin as reporter signal due to its intercalating capacity (top) and DPV curves of decreasing concentrations of target miRNA and the corresponding calibration curve (bottom). Reproduced from [87]—Published by The Royal Society of Chemistry.

Graphene oxide (GO), a derivative of graphene, is obtained by the functionalization of graphene with oxygen groups (hydroxyl, carboxyl and epoxide) and presents good solubility although it has a reduced electron transfer capacity [81]. This functionalization enhances graphene selectivity towards anchoring biological recognition elements. In electrochemical biosensors, reduced GO (rGO), a derivative of GO, was firstly reported as electrode modifier by Zhou et al. in 2009 [82] for the sensing of DNA, showing increased electrochemical activity compared to graphite electrode. Since then, graphene and its derivatives have been incorporated into different types of biosensors, including AD biosensors. ApoE4 gene biomarker was detected taking advantage of curcumin-graphene quantum dots (QDs) as dual electrochemical and fluorescence platform for the modification of indium tin oxide (ITO) electrodes [83]. Curcumin, also known as Diferuloylmethane (C_21_H_20_O_6_) is an antioxidant polyphenol that has been used for clinical purposes due to its anti-inflammatory, antibacterial and anti-cancer activity among other properties. In that work, curcumin coated graphene QDs were used as electrode modifiers (Figure 2A). More precisely, a transparent ITO electrode was coated successively with graphene quantum dots (GQD) and electro-polymerized curcumin and then, the probe DNA was immobilized upon the resultant CU-GQD-ITO platform by means of malonic acid and 1-ethyl-3-(3-dimethylaminopropyl) carbodiimide (EDC)/N-hydroxysuccinimide (NHS) chemistry. As curcumin is a fluorescence and electroactive molecule, the functionalization of the electrode with this substance was confirmed by recording the corresponding oxidation current, what facilitates the characterization of the sensor constructed. Finally, ApoE4 gene was added to test biosensors performance, showing a linear decrease in the amperometric response recorded after hybridization and quenching of curcumin signals, with a LOD of 16.7 fM. The incorporation of curcumin to this sensor is of great relevance since it facilitates the anchoring of recognition elements while providing a double recognition tool due to fluorescence capacity of this molecule.

Wu et al. [84] used a graphene-mesoporous silica hybrid (GSH) nanomaterial as electrochemical platform also for the detection of ApoE4 gene. Silicon-derived nanomaterials have gained attention as potential components for energetic or catalytic applications due to their biocompatibility, facile surface modification and electronic properties among others, been widely used in biosensing for increasing sensitivity, selectivity and detection capacity [85]. In that work, GSH generated by soft template-assisted reducing process, acted as reservoir for the accumulation of methylene blue, added to the electrode as electroactive reporter. Moreover, ferrocenecarboxylic acid was conjugated to the nanomaterial as built-in control molecule and probe DNA was also immobilized by a bifunctional cross-linker 4-Maleimidobutyric acid and N-hydroxysuccinimide ester onto the electrode. The “on-off” biosensor worked as follows: first, an assistant probe DNA, only partly complementary to the long DNA probe immobilized onto the electrode was hybridized, preventing the leakage of methylene blue. Then, the analyte (the full complemented DNA sequence) was added, competing with the assistant probe DNA for the binding to the DNA probe, destroying the double structure and facilitating the release of methylene blue and the consequent decrease in the differential pulse voltammetric (DPV) current measured. Even though this sensor does not provide a lower LOD (down to 10 fM) than that of previous works [83], the use of this “on-off” strategy seems to reduce systematic errors and to increase reproducibility.

With regard to AD related miRNAs, Congur et al. [86] used GO as electrode modifier to develop a RNA biosensor for the detection of miRNA-34a, a biomarker of AD and various types of cancers. Firstly, they let react electrochemically activated pencil graphite electrodes with EDC/NHS and then with GO for increasing surface area of the electrode and favoring immobilization of miRNA, generating single use GO sensors. This strategy provides a cost-effective and easy to use sensor device that requires the use of less chemical reagents, all important qualities for the transferability of the device. Concentration of miRNA-34a was measured by electrochemical impedance spectroscopy (EIS) on phosphate buffer saline (PBS), showing a LOD of 261.7 nM.

Another example of miRNA detection is the work performed by Azimzaeh et al. [87] for the detection of miRNA-137 using a screen-printed carbon electrode (SPCE) modified with electrochemically-reduced GO and gold nanowires for enhancing the sensitivity of the biosensor and facilitating the immobilization of single stranded DNA (ssDNA) probe (Figure 2B). As electrochemical tag, doxorubicin was employed, due to its capacity to get intercalated in double stranded oligonucleotides [88] and also its electroactive properties, so that correlating directly to the concentration of hybridized biomarker. Doxorubicin concentration was recorded by DPV showing a detection limit of 1.7 fM and a good selectivity in human serum samples, which represents a notable increase compared to similar works [86], what seems to indicate that the use of doxorubicin as reporting signal helps to increase sensitivity.

Further studies for the detection of DNA and RNA AD biomarkers by using other nanomaterials are gathered at Table 2 [89].

#### 4.1.2. Nanomaterials as Labels

Some nanomaterials have intrinsic properties that facilitate their use as electrochemical labels, which is a desirable characteristic for reducing time and complexity of measurements. Among all the nanomaterials, AuNPs have been the most extensively studied, as they are easy to synthesize and bioconjugate with antibodies [91]. Owing to their high surface-to-volume rate in particles between 1–100 nm and high surface energy, they favor the immobilization of biological recognition elements [92].

AuNPs, alone or in combination with other molecules, have been extensively used as signal reporters for AD biomarkers detection, thanks to their facility for anchoring biological elements and their electroactive and electrocatalytic properties.

**Figure 3:**
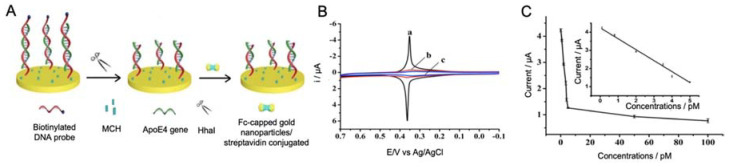
(**A**) Schematic representation of an electrochemical biosensor for the detection of ApoE4 gene using Fc-capped gold nanoparticles conjugated with streptavidin as labels and biotinylated DNA as capture probe. The biosensor principle is based on the specific cleavage of HhaI to double stranded DNA helix. 6-mercaptohexanol (MCH) is added as blocking agent. (**B**) CV responses of (a) biotinylated probe after hybridization with 50 pM ApoE4 gene, (b) same biotinylated probe after the addition of HhaI restriction enzyme and (c) unbiotinylated probe after hybridization with 50 pM ApoE4 gene; and (**C**) representation of the dependence between current (µA) and different concentrations of ApoE4 gene, where the insert represents the linear part between 0.1 and 5 pM. Reprinted by permission from: Springer Nature, Microchimica Acta, [90], 2018.

Lu et al. [90] developed a sensitive DNA sensor for the detection of ApoE4 gene using streptavidin-modified ferrocene capped AuNPs (Fc-AuNPs) as labels (Figure 3). By using a gold electrode modified with biotinylated specific oligonucleotides, ApoE4 gene was specifically trapped, generating a double chain DNA helix with the sequence of nucleotides GCGC. Such specific sequence is the cleavage site of the enzyme HhaI, a restriction enzyme that was used for increasing the specificity of the biosensor. Therefore, if the immobilized oligonucleotides hybridize with ApoE4 gene, GCGC sequence was generated in the middle of the double helix and by the addition of HhaI, the biotinylated fraction of the double stranded DNA was released, preventing the binding of Fc-AuNPs. Thus, the ApoE4 sequence could be discriminated against other ApoE sequences by EIS measurement with a LOD of 0.1 pM, which, although being low value, does not improve the LODs obtained with previous strategies.

### 4.2. Peptide Biomarkers

Aβ peptides and their different aggregated forms (oligomers, fibrils or plaques) are considered the main pathological hallmark of AD. From all Aβ peptides, Aβ(1-42) has been deeply investigated as it is more prone to aggregate thus being an important biomarker. For that reason, peptides are without no doubt the most investigated AD analytes in electrochemical biosensors. Below are described the main electrochemical biosensors for the detection of AD related peptides.

#### 4.2.1. Nanomaterials as Electrode Modifiers

- Gold nanoparticles

Aβ(1-42) peptide was detected by Wu et al. [93] using an EIS immunosensor based on an anodic aluminum oxide layer with a honeycomb-like surface as template for the electrodeposition of a gold film and over it AuNPs, used for the immobilization of monoclonal antibodies, specific only for the C-terminal end of Aβ(1-42) peptide, via 11-mercaptoundecanoic acid and EDC/NHS. The use of this nanomaterial may notably increase the surface area of the biosensor, so that increasing the amount of analyte captured. EIS was performed using Fe(CN)_6_^3−/4−^ as electroactive indicator for the determination of Aβ(1-42) peptide at different concentrations in buffer solution, obtaining a LOD of 22.2 fM. Increasing concentrations of Aβ(1-42) peptide revealed the appearance of aggregates examined by scanning electron microscopy (SEM) and atomic force microscopy (AFM).

Using a similar approximation Carneiro et al. [94] developed a label-free immunosensor for the detection of Aβ(1-42) peptide by using in this case a gold electrode modified with a mercaptopropionic acid monolayer and electrodeposited AuNPs. By square-wave voltammetry (SWV) and EIS using Fe(CN)_6_^3−/4−^ as electroactive indicator, the biosensor showed a LOD of 1.15 pM, although sensor selectivity and performance in real samples were not tested. LOD reached by this sensor is lower than the one exposed by Wu et al. [93], what may suggest that the use of a mercaptopropionic acid monolayer is not as effective as a gold film for the immobilization of AuNPs, although the selected electrode should also have an influence in the overall performance.

**Figure 4:**
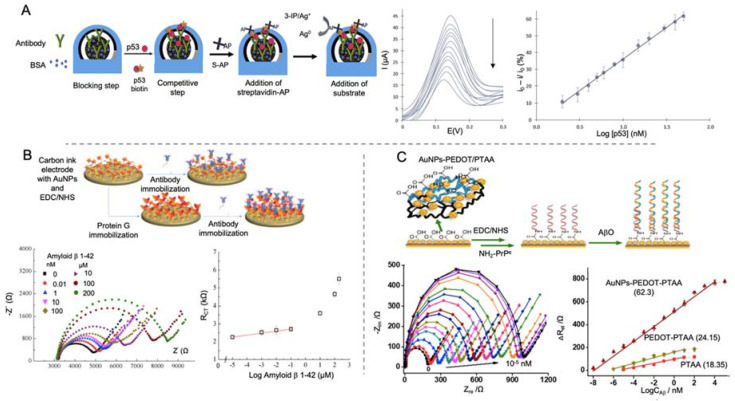
Electrochemical biosensors for the detection of AD peptides using AuNPs as electrode modifier. (**A**) Schematic representation of a competitive immunosensor for the detection of p53 peptide using streptavidin labelled alkaline phosphatase (S-AP) as electrochemical signal (left), accompanied by LSV voltammograms of different p53 concentrations and the corresponding calibration curve (right). Reprinted from [95]. Copyright (2020) with permission from Elsevier. (**B**) Schematic diagram of an immunosensor for the detection of Aβ peptide using different strategies; anchoring antibodies directly to the electrode by EDC/NHS or using protein G for the immobilization (top) and impedance curve of different concentrations of Aβ detected using antibodies immobilized by protein G and the associated calibration curve representing electron transfer resistance (RCT) vs log Aβ concentration (bottom). Reprinted from [99]. Copyright (2015) with permission from Elsevier. (**C**) Representation of a biosensor construction for the detection of Aβo using AuNPs and poly(thiophene-3-acetic acid) (PTAA) for the immobilization of PrPc receptor (top) and the Nyquist plot of different concentration of Aβo and the corresponding calibration curves (bottom). Adapted with permission from [100]. Copyright (2019) American Chemical Society.

Amor-Gutiérrez et al. [95] developed an immunosensor for the detection of unfolded p53 in blood samples (Figure 4A). Using a SPCEs modified with AuNPs for the immobilization of monoclonal antibodies against p53, they developed a competitive biosensor able to detect unfolded p53 by linear sweep voltammetry (LSV), with a LOD of 0.05 nM. The competitive assay consisted in the incorporation on the biosensor of biotinylated and non-biotinylated p53 for its specific detection by the immobilized antibodies. After that, streptavidin labelled with alkaline phosphatase was first added to the electrode for the binding to only biotinylated p53. By adding 3-indoxyl phosphate and, afterwards, silver nitrate solution, alkaline phosphatase catalyzed the enzymatic reduction of silver and generated the electrochemical signal by the anodic stripping of the produced Ag^0^. One of the main strengths of this work is the evaluation of the performance of the biosensor generated in real blood samples of patients with MCI and AD, showing no significant differences with ELISA results.

AuNPs or derivatives have also been widely used in sandwich immunoassays, that allow a double recognition of the biomarker detected. For AD biomarkers identification, several sandwich-type immunosensors using AuNPs are reported in Table 3 [96,97]. It is worthy to highlight that selectivity studies are missing in some of these works, although it is countered by the analysis of real samples.

But sandwich-type sensors not necessarily use only antibodies, since aptamer-based sandwich biosensors are also employed. Zhou et al. [98] built an aptamer-based biosensor for the detection of Aβo using AuNPs and Cu-MOFs (AuNPs/Cu-MOFs) nanocomposites as labels and gold nanoflowers (AuNFs) as electrode modifiers. AuNFs acted as immobilization platform for the primary aptamer while increasing the sensitivity of a glassy carbon electrode (GCE), reaching a LOD of 0.45 nM. Even though the flower-like structure of AuNFs should seem an advantage as it increases surface area, it does not represent an improvement in the LOD compared to previous works [93].

Affinity proteins, considered as noncatalytic and nonimmune generated proteins, are also used as recognition element, as they are able to interact with some molecules in a similar way that antibodies or enzymes do [115,116]. Lien and co-workers [99] developed an impedimetric immunosensor for the detection of Aβ peptides using carbon printed electrodes modified with protein G. For evaluating the effect of the electrode modification, they proposed a three-electrode immunosensor, each one with a different surface chemical modification (Figure 4B). First of them was the bare electrode functionalized by the monoclonal antibody against Aβ peptide by means of 1-pyrenebutanoic acid succinimidyl ester. For the second and third ones, they modified the electrode successively with AuNPs, generated by electrodeposition and then with a self-assemble monolayer (SAM) of 16-mercaptohexadecanoic acid (MHDA) for the further attachment of monoclonal antibodies. In the third electrode, protein G was immobilized on the surface before the addition of the antibody in order to orientate the latter. This modification helped to lower the LOD to 0.57 nM which is a notable reduction compared to bare electrode (2.04 μM) and protein G free electrode (2.65 nM) but not enough for reaching the lowest values seen in Aβ peptides detection.

Qin et al. [100] developed an impedance biosensor for the detection of Aβo by using cellular prion protein (PrPc) peptide as bioreceptor immobilized on to a gold electrode (Figure 4C). PrPc is a cell-surface glycoprotein that its conversion in an altered isoform is related with neurodegenerative infectious prion disease and it has been also postulated as receptor for Aβo [117]. In this biosensor, AuNPs embedded in a layer of poly (3,4-ethylene dioxythiophene) (AuNPs-PEDOT) and poly(thiophene-3-acetic acid) were successively electrodeposited onto for further anchoring of PrPc by EDC/NHS chemistry. Aβ was detected by EIS measurement in Aβ solutions and animal real samples, showing a LOD of 10^-2^ fM. This value is the lowest collected in this review for Aβ detection. This could indicate that the use of specific proteins, like PrPc could be an advantageous strategy for improving the detection of biomolecules with a high sensitivity and selectivity.

- Carbon nanotubes

CNTs are nanowires constituted by sp2 carbon atoms arranged in hexagons that were first observed in 1952 by Radushkevich and Lukyanovich [118]. Physical and chemical conditions during chemical synthesis lead to the formation of different types of CNTs listed by the number of walls they have, going from one (single-walled carbon nanotubes (SWCNTs)) to multiple walls (multi-walled carbon nanotubes (MWCNTs)) [119]. CNTs have been acknowledge for their electrochemical properties, including an enhanced electron transfer capacity, and also for their outstanding physical properties, making them ideal materials for biosensing [120]. In this context, Moreira et al. developed a biosensor for the detection of Aβo using CNTs and molecular imprinted polymers (MIPs) [102].

In general, biomarkers may be detected by the use of biological recognition elements as antibodies, aptamers or specific proteins. Alternatively, these recognition elements could be created artificially for mimicking these molecules with a high selectivity and versatility. MIPs, defined as synthetic polymers generated by polymerization in the presence of a template, have been thoroughly used in biosensing due to its engineerability [121,122].

**Figure 5:**
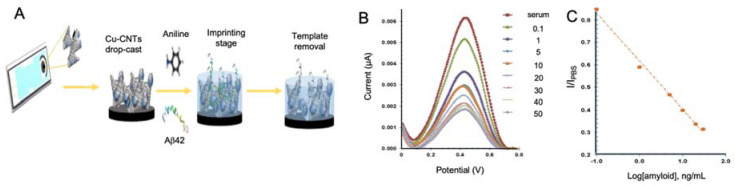
Electrochemical biosensors for the detection of AD peptides using CNTs as electrode modifier. (**A**) Representation of the generation of a MIPs for the detection of Aβ peptide using electropolymerized aniline and Cu-CNTs as electrode modifier, (**B**) SWV measurements of the modified electrode at different Aβ concentrations in human serum samples and (**C**) the corresponding calibration curve. Reprinted from [102]. Copyright (2018) with permission from Elsevier.

In AD biomarkers detection, MIPs were first used for the detection of Aβo by Moreira et al. [123] using α-cyclodextrin as polymeric matrix, achieving a LOD of 44 pM. In a subsequent study [102], the same group improved the performance of the biosensor by using aniline as polymeric matrix and CNTs modified with copper oxide nanoparticles as conductive substrate for improving electrocatalytic activity and electron transferability (Figure 5). Aβo detection was performed by cyclic voltammetry (CV) and SWV in artificial serum samples with a notable improvement in the performance compared to its previous work, reaching a LOD of 88.6 fM.

- Nanomembranes

Nanomembranes are structures of 1–100 nm thickness and a large of at least two magnitude orders more than their thickness. They have been implemented as scaffolds for nanoparticle synthesis or used in electrochemical devices as they endure repeated elastic deformation [124]. Nanomembranes are commonly made from organic polymers, like cellulose nitrate, polyacrylonitrile or polyvinyl as they offer a high selectivity and high fluxes [125]. They are often used forming nanocomposites, introducing two or more phases that contribute to enhance the functionality of the final structure, achieved by lamination, patterning, surface sculpting or introduction of nanoparticles [126].

Nanomembranes have been used for AD biosensing by Wustoni et al. [103] who implemented for the first time the combination of pristine isoporous membranes from the block copolymer poly(styrene-b-4-vinylpiridine) and organic electrochemical transistors (OECT) for the building of an immunosensor, in this case for the detection of Aβ peptide aggregates. OECTs are a type of transistor composed of a channel of an ion-permeable organic material through which an electrolyte solution is injected by the application of a voltage to the gate electrode [127]. By the immobilization of a biological recognition element inside the channel or the electrode gate, it is achieved the detection of specific proteins as its binding inside the channel partially blocks the ionic current, reducing the electrochemical signal recorded. However, the main drawback of this technology is that the immobilization of biological recognition elements may change the channel surface or even lead to electronic material degradation. To overcome this issue, Wustoni and coworkers placed, between the channel and the electrolyte solution, a 50 nm pore size pristine nanomembrane functionalized with Congo red as it has a strong affinity to a cross-β structure of Aβ aggregates, conferring the biosensor with a high specificity. As Aβ aggregates are bigger than the pore size of the membrane they block the ion transport in a concentration dependent manner, thus constituting a novel design of electrochemical immunosensors. The nanomembrane-based sensor detected Aβ aggregates with a LOD of 2.21 pM. Even though this value is not sufficiently low compared to other works, the implementation of nanomembranes in biosensing opens the way to its use in multianalyte detection.

- Nickel ferrite nanoparticles (NiFe_2_O_4_)

Nickel ferrite nanoparticles (NiFe_2_O_4_ NPs) are magnetic materials with a high resistance, permeability and saturation magnetization that have been highly implemented as gas sensors [128].

**Figure 6:**
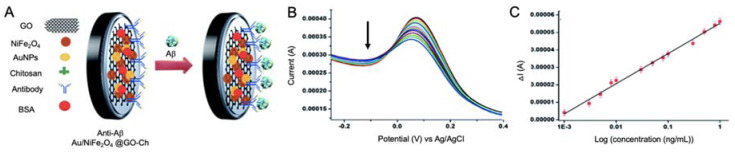
(**A**) Schematic representation of an immunosensor for the detection of Aβ peptide using GO, chitosan, AuNPs and NiFe_2_O_4_ NPs as electrode modifiers for the immobilization of antibodies and improving sensitivity; (**B**) DPV response of the biosensor at different Aβ peptide concentrations, revealing the decrease in the signal recorded while concentration is increased and (**C**) calibration curve showing linearity. Reproduced from [104]—Published by The Royal Society of Chemistry.

In the work performed by Devi et al. [104], NiFe_2_O_4_ NPs were used to develop a label-free immunosensor for the detection of Aβ peptide by using a GCE in combination with GO, chitosan and AuNPs (Au/NiFe_2_O_4_ NPs) (Figure 6). CV was used to evaluate the effect of the Au/NiFe_2_O_4_ NPs incorporation, showing an increased surface area and conductivity, while performance of the biosensor was evaluated in real CSF samples by DPV and EIS measurements, revealing a LOD of 0.66 pM.

#### 4.2.2. Nanomaterials as Labels

- Gold nanoparticles

**Figure 7:**
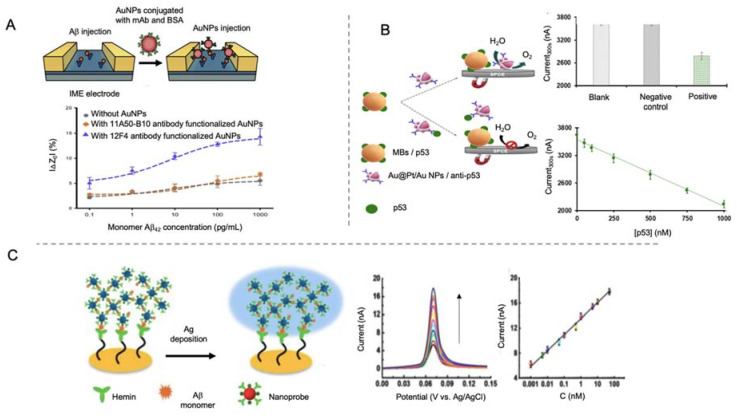
Electrochemical biosensors for the detection of AD peptides using AuNPs as labels. (**A**) Schematic representation of interdigitated microelectrode (IME) for the impedance detection of Aβ using AuNPs as labels (top) and the impedance curve with and without AuNPs and with different antibodies (bottom). Reprinted from [106]. Copyright (2020) with permission of Elsevier. (**B**) Schematic representation of a competitive immunosensor for the detection of unfolded p53 using core@shell Au@Pt/AuNPs for the catalysis of WOR and magnetic beads as immobilization platform. At the right, the analytical signal recorded for solutions without protein (blank), containing human IgG (negative control) and containing p53 peptide (positive); and the calibration curve for p53 concentrations between 50 nM to 1000 nM (bottom). Adapted with permission from [107]. Copyright (2020) American Chemical Society. (**C**) Schematic representation of a biosensor for the detection of Aβ monomer using hemin as bioreceptor and AuNPs and Ag deposition as electrochemical signal. At the right side the LSV curves of the biosensor performance at different Aβ monomer concentrations and the corresponding calibration curve. Republished with permission of Royal Society of Chemistry, from [110]; permission conveyed through Copyright Clearance Center, Inc.

AuNPs have also been used as labels for AD peptide biomarkers sensing [105,106,107,108,109,110,111]. Yoo et al. [106] designed a sandwich-type immunosensor for the detection of Aβ peptide by using an antibody-modified interdigitated microelectrode and AuNPs conjugated with antibodies as labels for signal amplification (Figure 7A). By measuring impedance changes in the presence of Aβ peptides, they obtained a LOD of 22.15 fM, what indicates that the use of AuNPs as labels for Aβ peptide is as valid as the use of AuNPs as electrode modifiers [94].

AuNP tags in combination with the use of magnetic bead platforms have also been employed for AD biomarkers detection. Magnetic micro- and nano-particles have been notably used for biomedical purposes, as drug delivery or hyperthermia, due to their unique properties, which include biocompatibility and superparamagnetic behavior of small magnetic nanoparticles [129,130]. In biosensing field, magnetic particles have become interesting tools especially as platforms for the immobilization of biological recognition elements, as their magnetic behavior allows the analyte pre-concentration and separation from complex samples, minimizing matrix effects. Taking advantage of magnetic bead platforms, Iglesias-Mayor et al. [107] proposed an immunosensor for the detection of unfolded p53 peptide using bifunctional core@shell Au@Pt/Au NPs as labels (Figure 7B). Au@Pt/Au NPs were synthesized by Pt deposition on AuNPs surface and the subsequent galvanic replacement reaction for the substitution of some of the Pt atoms by Au. These Au protuberances were used as anchoring element for the immobilization of antibodies. The electrocatalytic activity of Au@Pt/Au NPs toward the water oxidation reaction (WOR) allowed their sensitive chronoamperometic detection at neutral pH. This represented a high advance beyond the state of the art, since the measurements are performed in the same medium of the immunoassay, without the need of additional reagents. The immunosensor presented a LOD of 66 nM and was able to detect unfolded p53 in cognitively healthy subjects.

Aptamer-antibody sandwich sensors have also been investigated in order to overcome the lack of sensitivity and transferability to commercial products of aptamer-based biosensors alone, exhibiting an improved sensitivity and specificity compared to the latter [131]. This is the case of Zhou et al. [108] that used carboxyl graphene as electrode modifier for the immobilization of an antibody against Aβ oligomers using EDC/NHS chemistry and AuNPs functionalized with thionine as labels. The reduction process of thionine was electrochemically monitored for amplifying the signal recorded by DPV, providing a LOD of 100 pM. The use of thionine as electrochemical target seems to be less effective than AuNPs alone, as it was the case of Yoo et al. [106]. In this case, the difference between the analytical methods followed (impedance changes and DPV) may also be a determining factor that should be taken into consideration.

Moreover, affinity proteins were evaluated as recognition element in AD peptide detection. This is the case of gelsolin, a secretory protein present intracellularly and in CSF or plasma, that interacts with Aβ monomers extracellularly [132]. Yu et al. [109] developed a sandwich type biosensor using gelsolin as recognition element and AuNPs functionalized with gelsolin and horseradish peroxidase (HRP) as labels. The sensing principle was based on the recognition of Aβ(1-40) and Aβ(1-–42) peptides by gelsolin that was both bonded to the electrode and to the label. The HRP incorporated to the label catalyzed the oxidation of 3,3′,5,5′-tetramethylbenzidine in the presence of H_2_O_2_, generating a electrochemical signal measured by DPV. The use of gelsolin instead of other recognition element displayed satisfactory sensitivity in normal and AD rat brains even though the LOD was lower than previous revised studies (28 pM).

In a different approach, Ding et al. [110] used hemin as biological recognition element, taking advantage of its affinity to Aβ peptides. In this work, a gold microelectrode was used as template for the immobilization of hemin through cysteamine and EDC/NHS (Figure 7C). Polyethyleneimine (PEI) covered AuNPs were used as labels by functionalization of hemin and Cu^2+^ (Cu^2+^-PEI/AuNPs-hemin). The presence of Aβ peptides produced an aggregation network of Cu^2+^-PEI/AuNPs-hemin nanoprobes through specific Cu^2+^-Aβ-hemin coordination. Such aggregates were deposited on the microelectrode by joining their copper ions to the hemin groups of the electrode, and then generating active sites for the deposition of silver, which was finally detected by the well-known anodic stripping analysis. The main objective of depositing silver instead of using AuNPs directly was to amplify the signal, a purpose that, considering that the LOD obtained in this work was of 0.2 pM, was not completely reached compared to other works.

In a different approach, Xia et al. [111] developed a biosensor for the detection of Aβo based on the use of PrPc peptide as recognition element and AuNPs as labels. In this case, the affinity of AuNPs to peptides was explored, as they tend to form aggregates with free peptides, increasing the EIS signal detected. Once Aβ peptide was anchored to PrPc, AuNPs were added, so that they formed aggregates with free peptides anchored to the gold electrode through MCH. Performance of the biosensor was first evaluated using human chronic gonadotropin as model analyte and then implemented for blood serum to test sensitivity and selectivity against AD biomarkers, showing an extreme selectivity to Aβo even in the presence of Aβf and Aβ monomers and a LOD of 45 pM. If we compare this sensor with the one developed by Qin et al. [100], the LOD obtained is higher even though in both cases PrPc is used as recognition element. This shows that the detection strategy chosen, and the role of the nanomaterials are a fundamental part of the device generated.

- Silver nanoparticles

In a minor extent, AgNPs have also been used as tags in electrochemical biosensing [133]. AgNPs have marked properties, including catalytical activity and large surface area, like AuNPs have, but, in comparison, they have easier electrochemical oxidation capacity and higher extinction coefficient. However, they are instable and difficult to be functionalized, thus they have been rarely used for biosensing.

In AD, Xia et al. [112] proposed a biosensor for the detection of Aβo using the same principle than in their previous work with AuNPs [111] but in this case employing AgNPs modified with adamantine (for colorimetric testing) as labels. The AgNPs detection through the solid-state Ag/AgCl reaction allowed to achieve a LOD of 8 pM, lower than the reported for AuNPs.

You et al. [113] developed a sandwich-type aptamer-based biosensor for the detection of Aβo using a nanocomposite of AgNPs and silica nanoparticles (SiO_2_@AgNPs) as label. Detection of Aβo was performed by using MIPs, generated over a GCE functionalized with GO and AuNPs for improving electrical conductivity and surface-area rate, by using a polymer precursor solution composed of methacrylic acid, 1,3-diallylurea, divinylbenzene and N, N′- azo-bis-(2,4-dimethyl)valeronitrile. The voltammetric detection of AgNPs, amplified by the nanocomposite, allowed the determination of the biomarker in human samples with acceptable relative standard deviation (RSD) (2.5–9.8%) and a LOD of 0.27 pM.

- Metal-organic frameworks (MOFs)

MOFs are three-dimensional (3D) porous coordination polymers formed by the bridging of inorganic components with organic ligands [134], and have deeply been used in gas storage, chemical sensing, catalysis or drug delivery among other applications [135]. Even though the vast majority of MOF investigations have been focused on bulk systems, more recently, MOF nanoparticles have started to be synthesized as they have an improved bioavailability compared to bulk materials [136]. In electrochemical sensing, MOFs have been postulated as labels, as some of them exhibit a good electrochemical activity and high number of surface-active sites with enhanced enzyme-like catalytic activity [137]. However, they lack from a good electronic conductivity and electrocatalytic ability, so that they have been used in combination with other nanomaterials forming nanocomposites [138] or as electrochemical signal carriers.

**Figure 8:**
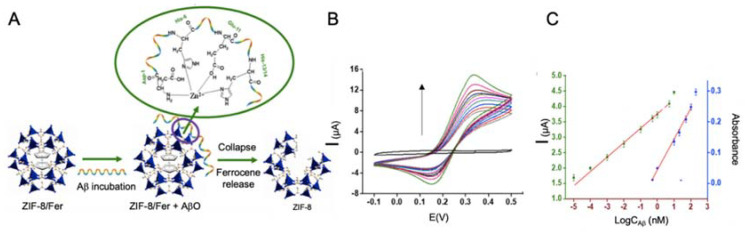
(**A**) Schematic representation of Aβo detection biosensor using ZIF-8 with ferrocene encapsulated as reporter, as it is able to release ferrocene specifically when Aβo is present due to its tendency to coordinate with Zn ions from ZIF-8. (**B**) CV curve of different concentrations of Aβ peptide and (**C**) the corresponding calibration curve. The biosensor is based on the aggregation capacity of Aβ peptide with Zn ions and the consequent destruction of ZIF-8 structure and the release of ferrocene. Adapted with permission from [114]. Copyright (2019) American Chemical Society.

In this line, Qin et al. [114] developed a biosensor for Aβo detection using Zn zeolite imidazole framework (ZIF-8) with ferrocene encapsulated as electrochemical signal (Figure 8). This biosensor was based on the contacting of ferrocene—ZIF-8 with Aβo, in a way that Zn ions of ZIF-8 tended to coordinate with Aβo causing the breakup of the ZIF-8 structure and the release of ferrocene. The supernatant of the solution was measured by CV to determine Aβo concentration with a LOD of 10^−5^ μM, much higher than that of the rest of the works revised for Aβo detection, what agrees with the lack of electronic conductivity of MOFs.

### 4.3. Protein Biomarkers

AD protein biomarkers are a spare target of investigation in early detection of this devastating disease. It is remarkable the earmarking of ApoE4 and tau protein in these sensors, without forgetting Aβ detection in its protein conformation. Minority biomarkers, as BACE-1 and α-1 antitrypsin have also been subject of study.

#### 4.3.1. Nanomaterials as Electrode Modifiers

- Gold nanoparticles

Liu et al. [139] proposed a sandwich-type electrochemical immunosensor for the detection of ApoE4, using an ITO electrode. For the modification of the ITO electrode they used a fractal gold nanostructure generated by electrodeposition, which due to its ramified conformation, it has a high surface-to-volume rate allowing the binding of a greater number of monoclonal antibodies against ApoE4. As label, in this case they used the enzyme HRP that catalyzes the oxidation of hydroquinone into quinine in the presence of H_2_O_2_. The reductive current generated was recorded by amperometry showing a detection limit of 8.78 pM.

**Figure 9:**
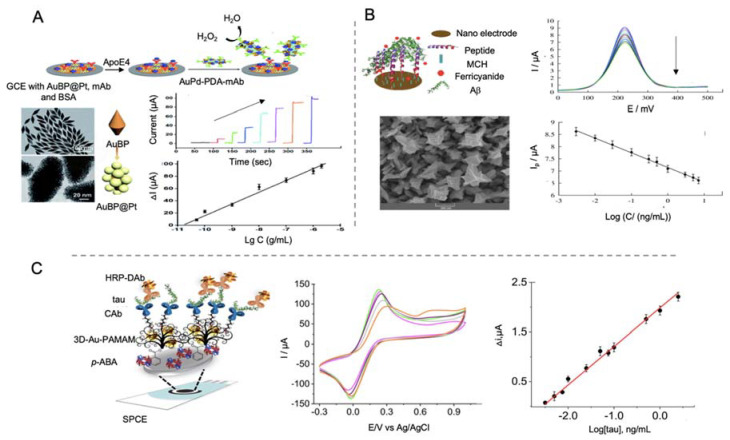
Electrochemical biosensors for the detection of AD proteins using AuNPs as electrode modifiers. (**A**) Schematic representation of a sandwich type immunosensor for the detection of ApoE4 using gold bipyramids coated with platinum as electrode modifier and polydopamine nanotubes doped with AuPd as labels (top), micrographs of SEM of AuBP, micrographs of TEM of AuBP@Pt, amperometric curves at different concentrations of ApoE4 protein and the corresponding calibration curve (bottom). Reproduced from [140] Published by The Royal Society of Chemistry. (**B**) Schematic representation of a biosensor for the detection of Aβ using a gold nanostructured electrode and ferrocene as reporter (top) and field-emission scanning electron microscopy (FESEM) of the gold nanostructures, DPV curves at different concentrations of Aβ protein and the corresponding calibration curve (bottom). Reprinted by permission from Springer Nature, Microchimica Acta [142]. Copyright (2019). (**C**) Schematic representation of a biosensor for the detection of tau protein using SPCEs modified with 3D-Au-PAMAM nanocomposites and using HRP enzyme as signal generator (left). At the right side CV curves at different stages of the functionalization process: 3D-Au-PAMAM-*p*-ABA-SPCE (magenta), GA-3D-Au-PAMAM-*p*-ABA-SPCE (green), CAb-3D-Au-PAMAM-*p*-ABA-SPCE (purple), blocked CAb-3D-Au-PAMAM-*p*-ABA-SPCE (grey) and HRP-DAb-tau-CAb-3D-Au-PAMAM-*p*-ABA-SPCE (orange) and calibration curve at different tau protein concentrations. Reprinted from [144]. Copyright (2020) with permission from Elsevier.

In a further work, the same group [140] developed also a sandwich-type biosensor for the detection of ApoE4 but in this occasion, using gold nanobipyramids coated with Pt nanoparticles deposited on a GCE/Au electrode for increasing conductivity and electroactive area (Figure 9A). After that, primary antibodies were self-assembled onto the transducer, obtaining a LOD of 0.45 pM, lower than the obtained in their previous work, what clarifies that the structure of the selected nanomaterial is important for the immobilization of biological recognition elements. Polydopamine nanotubes doped with AuPd were used as labels.

Negahdary et al. [141] explored an aptamer-based biosensor based on the electrodeposition of fern leaves-like gold nanostructures onto a gold electrode in the presence of polyethylene glycol 6000. Fern leaves-like gold nanostructures provided the electrode with a high surface area for the concentration and immobilization of the RNA aptamer, meanwhile polyethylene glycol 6000 was used for controlling size and shape of the nanomaterial. Detection of Aβ was performed by DPV using ferro/ferricyanide redox probe showing a LOD of 88.6 fM. In a subsequent work [142], the same group developed a biosensor also for the detection of Aβ using microporous gold nanostructures and Aβ peptide as bioreceptor (Figure 9B). In this case, the LOD was lowered to 44 fM, as the nanostructure allowed a good immobilization of Aβ peptide and the enhancement of the label diffusion.

Taking advantage of the combination of AuNPs and MOFs, Hang et al. [143] developed a biosensor for the detection of Aβ peptide using AuNPs for modifying the electrode and a flower-like zinc metal-organic framework (Zn-MOF) as label. AuNPs were incorporated both in the electrode and in the Zn-MOF to serve as anchoring platform for antibodies. The use of MOF nanomaterials as labels is revised in the corresponding section.

Tau protein has also been detected by Prof. Pingarrón group’s using AuNPs—poly(amidoamine) (PAMAM) dendrimer nanocomposite as electrode modifier [144]. In this work, first SPCEs were modified with AuNPs-PAMAM and electrografted *p*-aminobenzoic acid for the ulterior anchoring of antibodies through glutaraldehyde. HRP functionalized with specific antibodies was used for the electrochemical signal generation, upon de addition of hydroquinone and H_2_O_2_, recorded by amperometric detection with a LOD of 0.031 pM (Figure 9C). Biosensors performance was evaluated in human plasma samples and in brain tissue of healthy and AD diagnosed patients, what represents the main input of this work.

- Carbon nanotubes

**Figure 10:**
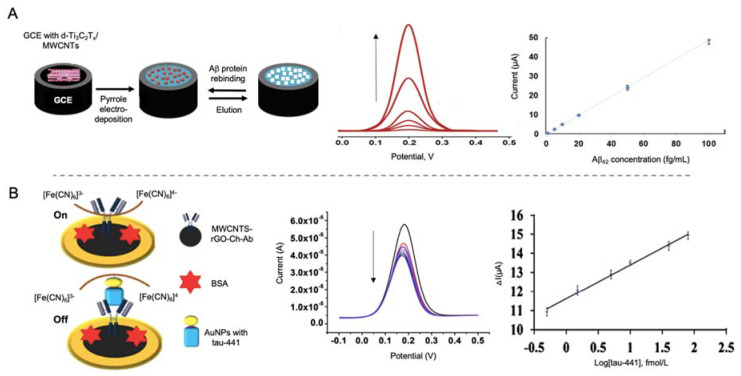
(**A**) Schematic representation of a MWCNTs modified electrode combined with MXene for the detection of Aβ protein using pyrrole as template for the generation of a MIPs as recognition platform (left) and the DPV curves at different concentrations of Aβ protein and the corresponding calibration curve (right). Reprinted from [145]. Copyright (2020), with permission from Elsevier. (**B**) Biosensor for the detection of tau-441 using MWCNTs-rGo with chitosan as electrode modifiers for the immobilization of antibodies and AuNPs conjugated with tau-441 for signal amplification. The biosensor works with an on/off system based on the blocking of electron transference of [Fe(CN)_6_]^3−/4−^ in the presence of tau-441. At the right side the DPV curves of growing concentrations of tau-441 and the corresponding calibration curve. Reprinted by permission from Springer Nature, Microchimica Acta [147]. Copyright (2020).

MWCNTs have been used by Özcan et al. [145] in the development of a biosensor for the detection of Aβ protein using a combination of delaminated titanium carbamide MXene (d-Ti3C2TxMXene) and MWCNTs to modify a GCE electrode (Figure 10A). MXene are 2D materials formed by early transition metal carbides and/or carbonitrides generated by etching of A elements of MAX phases (M = Ti, V, Nb; A = elements from IIA and IVA; X = N and/or C). These materials are easy to fabricate and have a good electrical conductivity, so they are widely used as electrode materials [146]. In this work, d-Ti3C2TxMXene was combined with MWCNTs as they prevented the aggregation of Mxene. MIPs of pyrrole were generated as template for the detection of Aβ by DPV with a LOD of 44 aM, the lowest found in all the works revised in this review paper. This value seems to highlight, both the important role of CNTs as electrode modifiers and the utility of MXene as electrode construction material. This last point, as well as its ease of manufacturing, are of special concern with regard to commercialization of AD biomarker detection biosensors.

Tau-441 protein was detected by Li et al. [147] using MWCNTs and rGO as electrode modifiers and AuNPs for signal amplification (Figure 10B). The synergistic effect of MWCNTs and rGO provided a better electron transfer than with both nanomaterials alone and the binding of chitosan to MWCNTs by electrostatic forces allowed the anchoring through glutaraldehyde of the capturing antibodies. Tau-441 was conjugated to AuNPs through cysteamine for its capturing by the antibodies immobilized onto the electrode. When affinity recognition occurred, electron transfer of [Fe(CN)_6_]^3−/4−^ was blocked, reducing the electron transfer process measured by DPV, providing a LOD of 0.46 fM.

#### 4.3.2. Nanomaterials as Labels

- Gold nanoparticles

Shui et al. [148] developed a biosensor for the detection of tau-381 protein biomarker in human serum, using an antibody-aptamer based biosensor. In this case, cysteamine-stabilized AuNPs were used as probe for the amplification of the electrochemical signal recorded by DPV. Cysteamine is a stable aminothiol used in nanoparticles synthesis since, due to its -NH^3+^ terminus, provides nanoparticles with an external positive charge, preventing aggregation and facilitating the interaction with negatively charged oligonucleotides [149]. The use of AuNPs as signal amplifiers allowed a LOD of 0.42 pM. Human serum of AD patients was tested verifying the feasibility of the biosensor and its potential use as clinical test.

Using a bimetallic nanocomposite of polydopamine nanotubes doped with AuPd alloy, Liu et al. [140] developed an immunosensor for the detection of ApoE4, deeply described above. AuPd alloys were bio-conjugated with secondary antibodies and used as labels for catalyzing the reduction of H_2_O_2_ as electrochemical signal, although they achieved a LOD of only 0.45 pM. Even though the LOD may be improved, the use of AuPd alloys as catalytical labels favors its use directly in the same media as immunoreaction takes place, as it has been highlighted in above mentioned works [107], what reduces the analysis time.

- Metal-organic frameworks

**Figure 11:**
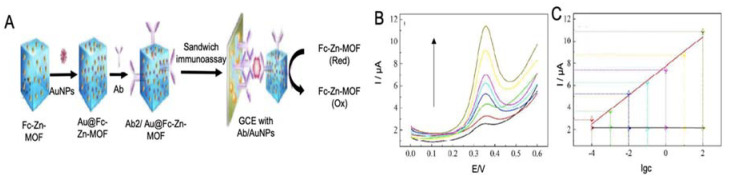
(**A**) Schematic representation of a sandwich type immunosensor for the detection of Aβ protein using AuNPs as electrode modifiers and Zn-MOF with ferrocene encapsulated as electrochemical signal and AuNPs conjugated for the immobilization of antibodies. The analytical signal was obtained by the oxidation of ferrocene, (**B**) the SWV curves at different Aβ protein concentrations and. (**C**) the corresponding calibration curve. Reproduced from [143]-Published by The Royal Society of Chemistry.

MOFs have also been used as labels for the detection of AD proteins due to their good electrochemical and catalytic activity. In the work performed by Hang et al. [143] Zn-MOF, functionalized with ferrocene and with AuNPs for the immobilization of antibodies, were used as labels for the detection of Aβ (Figure 11). The flower like structure of Zn-MOF allowed an enhanced electrochemical signal and allowed an upgraded immobilization of antibodies, which increased the performance of the biosensor, reaching a LOD of 6.6 fM.

- Cerium oxide nanoparticles

Cerium oxide nanoparticles (CeNPs) have interesting catalytic and electrochemical properties that make them a valuable tool for biosensing [150]. Moreover, their high electron-transfer rate and surface coverage make them an excellent co-immobilization material [151].

**Figure 12:**
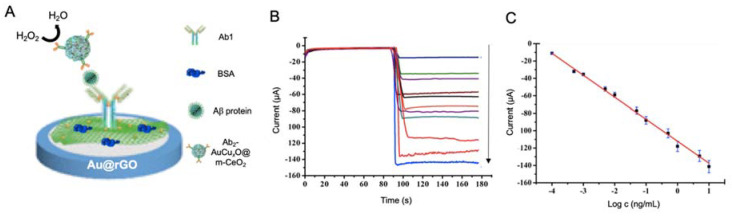
(**A**) Schematic representation of a biosensor for the detection of Aβ protein using Au@rGO as electrode modifier and anchoring platform of antibodies (Ab1) and Ab-AuCuxO@m-CeO_2_ as labels for the reduction of H_2_O_2_. BSA was incorporated as blocking agent. At the right, (**B**) current response curves of the biosensor and (**C**) the calibration curve at different Aβ protein concentrations. Adapted with permission from [152]. Copyright (2019) American Chemical Society.

Gao et al. [152] proposed an immunosensor for the detection of Aβ using a AuCuxO-embedded mesoporous CeO_2_ (AuCuxO@m-CeO_2_) nanocomposites as electrocatalytic label and AuNPs-functionalized with GO as electrode modifier (Figure 12). The role of m-CeO_2_ in the nanocomposite was serving as immobilization platform for the antibodies, as it tends to form bonds with carboxyl functional groups of the latter. In a complementary way AuCuxO served as catalytic platform, as CuxO has a tendency for the reduction of H_2_O_2_ to H_2_O while Au decreases the activation energy required for the reaction. By measuring this process, Aβ protein was successfully detected with a LOD of 7.97 fM.

- Quantum dots

QDs are inorganic semiconductor nanocrystals extensively used in optoelectronics. Nonetheless they are also exploited as electrochemical labels thanks to the presence of electroactive metals on their composition [153].

Medina-Sánchez et al. [154] developed a sandwich immunosensor for the detection of ApoE by using QDs as labels. In this work, cadmium-selenide/zinc-sulfide (CdSe@ZnS) QDs were used as labels while tosylactivated magnetic beads were used to modify polydimethylsiloxane (PDMS)-polycarbonate (PC) microfluidic chip integrated into screen-printed electrodes. Firstly, magnetic beads were functionalized with antibodies and anchored to the electrode by using a neodymium magnet. Then, ApoE in different concentrations was incorporated followed by the addition of biotinylated antibodies. Streptavidin modified QDs were then flushed and used for the electrochemical measurement of Cd^2+^ reduction and re-oxidation by SWV, providing the determination of ApoE in a LOD of 0.37 nM, higher than values reported in previous works. Selectivity and performance in real samples were also evaluated showing a good correlation between the results by the biosensor and standard techniques, what it is a valuable probe of the viability of the device.

- Iridium oxide nanoparticles (IrO_2_ NPs)

IrO_2_ NPs have been applied in biosensing due to their excellent catalytic activity toward WOR and H_2_O_2_ reduction, what makes them suitable labels for detection at neutral pH [155]. Moreover, they present wide surface specificity for the immobilization of biological recognition elements [156] and a high biocompatibility [157].

**Figure 13:**
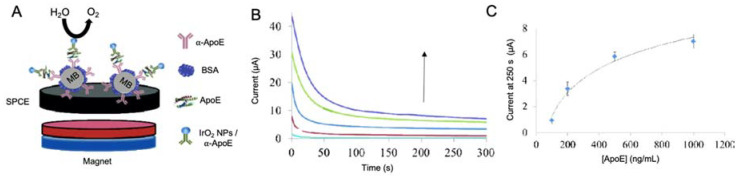
(**A**) Schematic representation of a sandwich type immunosensor for the detection of ApoE4 based on the use of magnetic beads platforms and IrO_2_ NP as labels for the catalysis of WOR. The use of MB allows the separation and purification of the process by the use of an external magnetic field. At the right, (**B**) chronoamperograms at different ApoE4 concentrations and (**C**) its corresponding calibration curve. Adapted with permission from [158].

ApoE was electrocatalytically detected by Rivas et al. [158] by using carboxylated magnetic microbeads as anchoring platform for the immobilization of antibodies and IrO_2_ NPs as labels (Figure 13). ApoE was detected by following the catalytic effect of IrO_2_ NPs toward the WOR in chronoamperometric mode, with a LOD of 1.99 nM, with reproducible results in human plasma samples. As it happens with the work developed by Liu et al. [140], the use of this kind of labels does not allow reaching low LODs, but the electrocatalytic properties of the nanomaterials help to simplify the measurement procedure.

Further examples of the use of other nanomaterials as labels for AD protein sensing are revised in Table 4 [45,46].

### 4.4. Neurotransmitter Biomarkers

Neurotransmitters are endogenous neurochemicals that enable neuron communication in order to maintain synaptic and cognitive function [159]. Minor changes in the synthesis or release of neurotransmitters have been related with neurological disorders like AD or Parkinson’s Disease, so that they have been proposed as suitable biomarkers for the detection and treatment of neurodegenerative disorders [49].

Recently, liquid chromatography, microdialysis or capillary electrophoresis, among others, have been used for neurotransmitter detection. However, these techniques are time-consuming, so they do not constitute an adequate screening method. Therefore, optical and electrochemical sensing have emerged as an alternative. But, even though a great number of biosensors have been developed for neurotransmitter detection, they have not only been applied to AD, being mainly focused on water and food safety purposes [160,161].

**Figure 14:**
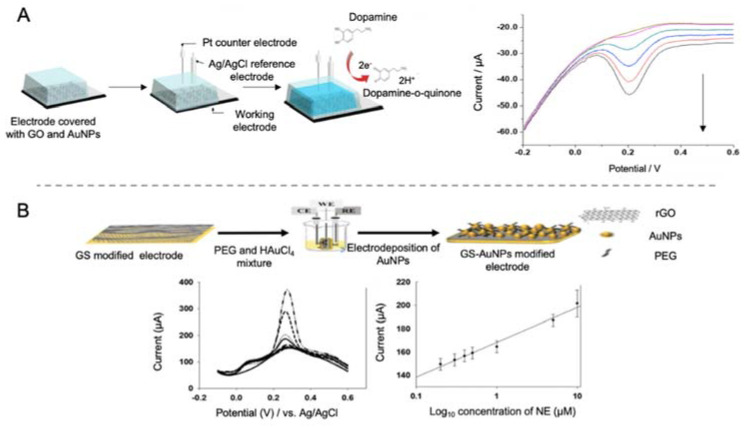
Biosensors for the detection of neurotransmitters. (**A**) Schematic representation of a biosensor for the direct detection of DA using GO and AgNPs by direct detection of the oxidation process of this molecule. At the right side, the DPV curves at different concentrations of DA. Reproduced with permission from [41]. (**B**) Schematic representation of a biosensor for the direct detection of NE by using a rGO and electrodeposited AuNPs modified electrode (top), accompanied with the DPV curves at different concentrations of norepinephrine and the corresponding calibration curve (bottom). Reprinted by permission from Springer Nature, Korean Journal of Chemical Engineering [42]. Copyright (2017).

For AD detection, Shin et al. [41] proposed a biosensor for the detection of DA, a neurotransmitter responsible for memory, behavior and movement. By using an ITO working electrode modified with GO and AgNPs (Figure 14A), they achieved a lower LOD compared to previous works that employed gold or carbon-based nanomaterials [162,163], improving biosensing performance at low DA levels. The electrochemical detection of DA was performed by direct detection of the oxidation process of this molecule by CV, DPV and amperometry. Ascorbic acid (AA) and uric acid (UA) were used for the evaluation of the biosensor selectivity, as they have similar oxidative potentials than DA and other neurotransmitters and is present in blood samples at higher concentrations [164]. The biosensor showed a specific response to DA at 0.2 µM levels.

DA has also been detected by Park et al. [165] using a gold electrode modified with a nanocomposite of rGO sheets and AuNPs. rGO sheets were used to improve the sensitivity of the biosensor due to their great conductivity while AuNPs where incorporated for improving electrocatalytic activity. Selectivity was achieved as π electron of rGO sheets tend to form π-π bonds with benzene rings of aromatic compounds as DA. However, other molecules with a similar structure as DA, like AA, may affect the selectivity of the biosensor. For that reason, the assay was performed at pH 7.4 at which AA exists as an anion while DA as a cation, thus facilitating the electrostatic interaction of DA with the electrode surface. DA was detected with a LOD of 0.098 μM in the presence of AA 400 μM.

Using a similar approach, Lee et al. [42] developed a biosensor for the detection of NE using rGO and AuNPs as electrode modifiers (Figure 14B). NE is a monoamine neurotransmitter highly related with neural diseases. However, the main drawback of NE detection is its similarity with AA and UA, as they have similar electrochemical oxidation potentials. In the biosensor developed in this case, detection of NE was achieved with a LOD of 200 nM in the presence of a concentration of AA 2000 times higher.

Another neurotransmitter highly studied for its relationship with AD is ACh. ACh is a neurochemical molecule used by cholinergic neurons, present in peripheral and central nervous system. It is involved in cognition, memory and movement, so it constitutes an important modulator of AD dysfunction [166].

Da Silva et al. [167] developed a biosensor for the detection of ACh using the enzyme acetylcholinesterase as receptor. To increase surface-to-volume rate and provide the biosensor with an appropriate immobilization matrix, they used Fe_2_O_3_ nanoparticles due to its strong absorption properties and electron-transfer capacity. In order to have an enhanced applicability, Fe_2_O_3_ nanoparticles were modified with films of poly (neutral red) generated by electropolymerization in the presence of ethaline deep eutectic solvents (DES) with the addition of acid dopants. Acetylcholinesterase was further immobilized by glutaraldehyde cross-linking. The feasibility of the biosensor was evaluated in synthetic urine samples by amperometric measurement showing a LOD of 1.04 μM. Further examples of biosensors for the detection of neurotransmitters using nanomaterials and developed in the last ten years are collected in Table 5 [168,169,170].

While it is true that neurotransmitters detection is an important parameter for AD detection, their use as biomarkers it is still not widespread, what is reflected in the number of biosensors that test neurotransmitters detection in real samples. Moreover, of special interest are the interferences with other substances as UA or AA, for what the approximation followed by Lee et al. [42] is the most promising for increasing selectivity. Regarding LOD, it should be highlighted that the obtained values are lower than those reported for other AD biomarkers. Regarding the nanomaterials, the use of graphene derivatives is much more widespread than for other AD biomarkers, what may be due to its tendency to form π-π bonds with aromatic compounds.

### 4.5. Oxidative Stress Biomarkers

The imbalance between free radicals’ generation and degradation in the body plays a significant role in the pathogenesis of cancer, autoimmune diseases and neurodegenerative diseases, including AD [43]. Most of the biological molecules that compose neurons can be oxidized due to mitochondrial dysfunction or inflammation, being both processes that occur during AD. As a consequence of an increase in the presence of reactive oxygen species (ROS) or other oxidative stress biomarkers [171] Aβ deposition and tau hyperphosphorylation are enhanced. When the levels of oxidative stress biomarkers are disbalanced in the brain, values in blood are also increased, facilitating their detection [50] and serving as early indicator of health issue. The main drawback of this biomarkers is the lack of specificity, as oxidative stress is associated to a wide variety of health issues.

**Figure 15:**
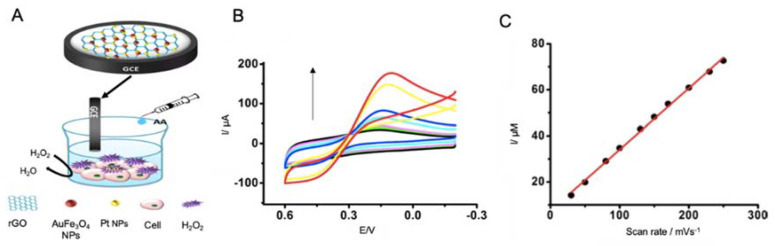
Electrochemical biosensors for the detection of oxidative stress biomarkers. (**A**) Schematic representation of a biosensor for the detection of H_2_O_2_ in culture media using a GCE modified with rGO, AuFe_3_O_4_ NPs and PtNPs, that is introduced in a cell culture under oxidative stress conditions provoked by the addition of AA to culture media. These conditions promote the release of H_2_O_2_ by the cells, indicating the oxidative stress suffered. (**B**) CV curves of different H_2_O_2_ concentrations and (**C**) the corresponding calibration curve. Adapted with permission from [172]. Copyright (2015) American Chemical Society.

Wang et al. [172] developed a biosensor for the detection of H_2_O_2_ as a potential oxidative stress biomarker (Figure 15). This biosensor was based on the use of a GCE functionalized with rGO blended with Au, Fe_3_O_4_ and platinum nanoparticles generated by electrodeposition. For in vitro detection, working electrode was introduced in cellular culture media, in which H_2_O_2_ was present as it was liberated by cells under oxidative stress conditions, provoked by the injection of AA. Reduction of H_2_O_2_ was measured by CV, providing a LOD of 0.1 μM, without the need of enzymatic labels, what notably reduced the analysis time.

Sekar et al. [173] developed a biosensor for the detection of H_2_O_2_ in human blood using zinc oxide-polyvinyl alcohol (ZnO-PVA) nanocomposites as electrode modifiers. ZnO was selected in this work as it has a high electronic conductivity and high catalytic activity. Functionalization with PVA provided a higher electrical conductivity compared to bare ZnO and the presence of -OH groups facilitated electron density on the biosensors surface. For the specific catalysis of H_2_O_2_ into H_2_O and O_2_, the enzyme catalase was immobilized in the electrode surface and the process was recorded by CV, allowing to reach a LOD of 9.13 nM. Following a similar approach, other studies have been developed for the detection of H_2_O_2_ and are revised in Table 6 [174,175,176].

H_2_O_2_ detection constitutes an interesting way of monitoring oxidative stress, what is nowadays of special concern specially for the study of AD disease as reflected in the application of the sensors described above in in vitro cell cultures. In any case, it is required further investigation on the clinical parameters of oxidative stress in AD patients before the implementation of this biosensors. However, the development of this technology and its study focused on AD is a sign of progress in this field.

### 4.6. Multianalyte Detection

The multifactorial profile of AD implies the contribution of proteins and genetic biomarkers to its etiology that conditions the development of the disease. All the above revised biomarkers contribute to the development of AD so that the evaluation of one biomarker alone do not constitute an accurate diagnosing tool. Moreover, there are at least five different subgroups of AD patients differentiated by the concentration of Aβ and tau levels in CSF, what affects the efficiency of medical treatments [177]. For that reason, the development of multianalyte biosensors has emerged as a prosper alternative for AD detection, as summarized in Table 7.

An initial approximation for this purpose is the identification, using the same biosensor, of different biomarkers. This is the case of de la Escosura-Muñiz et al. [178], that explored the detection of ApoE and Aβ peptide using magnetic beads as platforms of the immunoreaction and AuNPs tags. The electrocatalytic activity of AuNPs toward the hydrogen evolution reaction (HER) allowed the chronoamperometric detection of such biomarkers at clinically relevant levels in CSF, serum and plasma samples of patients suffering from AD. The performance of the biosensor was successfully evaluated, showing a LOD of 4.21 pM for Aβ peptide and 2.34 pM for ApoE.

In a similar approximation, Tau protein was detected by Serafin et al. [179] in combination with TAR DNA binding protein 43 (TDP-43), a protein involved in the evolution of several neurodegenerative diseases that has been found to form deposits in AD [180]. In this biosensor, AuNPs combined with PAMAM were used as scaffolds for the immobilization of antibodies to dual SPCEs, while HRP functionalized with antibodies was used for the amperometric detection of AD biomarkers using H_2_O_2_ / hydroquinone system as reporter, providing a LOD of 50 fM for tau-441 and 0.287 pM for TDP-43.

**Figure 16:**
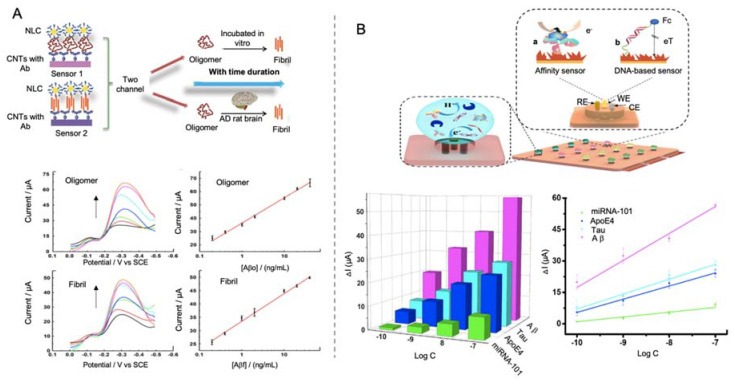
(**A**) Schematic representation of a biosensor for the combined detection of Aβo and Aβf in a two-channel system for monitoring Aβ aggregation among time using CNTs with antibodies as electrode modifiers and Poloxamer nanocarriers (NLC) as labels (top). DPV curves for different concentrations of oligomers and fibrils and the corresponding calibration curves (bottom). Adapted with permission from [182]. Copyright (2019) American Chemical Society. (**B**) Mini-pillar sensor of polydimethylsiloxane and gold nanodendrites for the detection of multiple AD biomarkers at the same time. Each hole of the biosensor constitutes a reservoir in which biomarkers interact with the recognition element producing an increase in the surface resistance in the case of affinity biomarkers (Tau, ApoE4 and Aβ) and reducing the electrochemical signal reported by ferrocene (Fc) in the case of DNA probes for miRNA-101 detection. At the bottom, the electrochemical response of different concentration of the AD biomarkers measured and the corresponding calibration curve (green: miRNA-101, blue: ApoE4, cyan: Tau, magenta: Aβ). Reprinted from [183]. Copyright (2020) with permission from Elsevier.

CNTs have been used by Yu et al. [181] for the identification of Cu^2+^ and Aβ monomers taking advantage of the affinity protein neurokinin B (NKB), that enhances Aβ aggregation capacity. In this competitive biosensor, Cu^2+^ and Aβ monomers were detected by using the specificity of NKB to Cu^2+^ and the Cu^2+^ tendency to form aggregates with Aβ, obtaining a more complete detection tool. First, a GCE was modified with SWCNTs functionalized with poly (diallyldimethylammonium chloride) (PDDA) and 2,2-azinobis-(3-ethylbenzthiazoline-6-sulphonate) (ABTS), as inner reference molecule and as sensitivity enlarger. NKB was coated on the ABTS-PDDA/CNTs electrode surface to form the complex [CuII(NKB)2]. After that, certain amounts of Aβ dispersed in Cu^2+-^ containing PBS where added, promoting the generation of aggregates and the release of Cu^2+^ from NKB. Quantification of Cu^2+^ and Aβ(1-42) was performed by comparing the reduction peaks at -0.12 V of Cu^2+^ and Aβ and at 0.58 V of the ABTS, obtaining a LOD of 0.04 µM for Cu^2+^ and 0.11 nM for Aβ. In a subsequent work, the same group [182] developed an immunosensor for the detection of Aβo and Aβf while monitoring the aggregation process (Figure 16A). For that purpose, a two-channel electrochemical system was constructed, with a specific antibody (against oligomers or fibrils) in each channel. Electrodes were functionalized with CNTs and polymer ion liquid to facilitate the electron transfer and the immobilization of the antibodies. Nanostructure lipid carriers made of poloxamer and functionalized with antibodies and thionine were used as labels, taking advantage of thionine as signal reporter. The principle of the sensor is based on the aggregation of Aβo into fibrils among time, as Aβo tends to get converted into Aβf reducing the signal corresponding to Aβo. The biosensor was tested in CSF and tissue samples of AD rats, constituting the first biosensors for the monitoring of Aβ aggregation among time. Yu and co-workers achieved in this case a LOD for Aβ detection of 2.2 pM for Aβo and 4.4 pM for Aβf.

In a more complete approximation, Song et al. [183] were able to detect four different AD biomarkers in the same platform (Tau, ApoE4, Aβ and miRNA-101) by using a mini-pillar sensor modified with gold nanodendrites (Figure 16B). This sensor was constructed by using polydimethylsiloxane as prepolymer and a template of polytetrafluoroethylene with holes of ~5 mm of diameter and ~1.65 mm of height. On each hole of the array they were embedded an Au working electrode, a Pt counter electrode and a reference electrode of Ag. After the construction of the mini-pillar polydimethylsiloxane electrode, gold nanodendrites were electrodeposited onto the working electrode for favoring the immobilization of both specific antibodies and DNA probes. This open-channel biosensor allowed the simultaneous sensing of trace amounts of different biomarkers, reaching LODs of 91.4 pM for miRNA-101, 1.29 fM for tau, 2.09 fM for ApoE4 and 1.9 fM for Aβ.

Even though LOD values are in some cases higher than the ones reached by other individual sensors, the ability to monitor the amount of different biomarkers in a single analysis is crucial for providing a more complete diagnosis and to reduce the measurement time and the number of samples required. This would reduce the number of invasive extractions needed to be performed on the patient. All of this constitute the way to obtain biosensor AD diagnostics close to the market requirements.

## 5. Conclusions

AD incidence is growing each day, and with it grows the social and economic burden associated to this devastating disease. Although there is not still a cure, an early detection of AD is crucial to delay as much as possible the appearance of degenerative effects and to allow family and carers to prepare themselves for the changes associated to it. Therefore, the development of an analytical device for the detection of early biomarkers of AD is required. In this context, the main obstacle to overcome is achieving good sensitivity and selectivity, as CSF and blood serum are complex samples that could interfere in the outcome of measurements. In this field, notably most of the biosensors revised in this work have evaluated the selectivity of the sensor against other widely spread biomolecules such as HSA, BSA or glucose. Those that do not separately evaluate selectivity against concrete biomolecules, mostly test biosensors performance in real samples.

Furthermore, efficiency and long-term stability need also to be evaluated as they are essential for the use of biosensors as screening methods. For that purpose, nanomaterials have been implemented in biosensing, in association with the outstanding progress made in nanomaterials synthesis and functionalization. Within this framework, nanomaterials have been implemented in biosensors as electrode modifiers, being gold and carbon-based nanomaterials the most widespread, specially MWCNTs that have allowed to achieve the lowest LOD found in the biosensors included in this review (10^−2^ fM). The modification of transducers with these nanostructures let a better electrochemical performance as its use improves conductivity, while favoring immobilization and orientation of biological recognition elements. In addition, the use of nanomaterials as labels, has provided an amplification of the signal, due to their electroactivity and electrocatalytic properties, that even though its use is more complicated, enables a higher sensitivity. In this field, again, the use of gold nanomaterials, alone or in combination with others, is massive, followed by the use of MOFs as reporter carriers. The use of electrocatalytic labels has also been a valuable improvement as it facilitates the detection of biomolecules at neutral pH, offering a more integrated sensing systems, also reducing the time of analysis which is of key relevance for meeting the market needs.

According to this, it should be noted that, in terms of LOD, the use of labels has not supposed an increased advantage in the amplification of the signal compared to the use of nanomaterials as electrode modifiers. Neither the use of nanomaterials as both electrode modifiers and labels has allowed to reach the lowest LODs. This highlights that there is much room for improvement in the implementation of combinations of nanomaterials for biosensing.

Surprisingly, lowest LODs have been achieved in biosensors using MIPs or recognition proteins, what suggests that the biological recognition element employed should be another parameter that should be taken into consideration.

Regarding the applicability of these biosensors for the detection of AD biomarkers highlights the great number of investigations related with the detection of Aβ peptide and its different aggregated forms, as it is still considered the main pathological hallmark of AD. However, it is interesting the increase in the detection of other biomarkers, such as ApoE4 and unfolded p53, that can shed light in the early diagnosis of AD. Moreover, the increased development of biosensor for the detection of multiple analytes is encouraging, as it supposes an advanced in the obtention of more complete diagnostic tools. However, many of the revised works do not evaluate final sensors performance in real human plasma or CSF samples of AD patients and even less compare the results with those provided by traditional techniques as ELISA. This point should be of special relevance to validate the applicability of the biosensors developed.

In any case, even though sensitivity and selectivity issues seem to be overcome, as fM and pM detection limits are reached, efficiency and long-term stability are not taken into account in many works, which is a problem for the transferability of the biosensors created. It is also a drawback the lack of evaluation of biosensors performance in real samples, that it is definitely necessary for evaluating biosensors effectiveness.

All these challenges need to be overcome if a commercial AD biosensor wants to be developed, and for that purpose these parameters should be assessed if we want AD early detection to become a reality.

## Figures and Tables

**Table 1:** AD biomarkers accepted and the role that they have in AD pathogenesis.

Biomarker	Pathogenicity in AD	Reference
Aβ peptide	Forms aggregates that accumulate extracellularly around neurons forming senile plaques, causing neurotoxicity and dementia.	[17,18]
Aβo	Aggregated form of Aβ peptide, present in CSF samples that produces cognitive impairment by inducing the depolarization of neurons’ membranes	[19,20,21]
Hyperphosphorylated tau	The abnormality phosphorylation of tau promotes its aggregation forming PHF, that form intracellular NFTs, neuropil threads and dystrophic neurons extracellularly accumulated near Aβ plaques.	[24,25,26,27,28]
ApoE4	Acts as a binding protein for Aβ, promoting the formation of β-sheets. It is the main biomarker related with early onset dementia.	[31,32]
Unfolded p53	It is present at nanomolar concentrations of Aβ peptide as a consequence of it but previously to the formation of notable Aβ plaques.	[36,37,38]
Neurotransmitters	They could interact with Aβo and minor changes on its synthesis and release could be associated to neurodegenerative diseases.	[39,49]
Oxidative stress	Induce by inflammation, Aβ formation and hyperphosphorylation of tau. Little changes in the brain could induce a disbalance in blood samples.	[43,50]
miRNAs	Short oligonucleotides that regulate the translation of mRNA of presenilins and APP genes.	[51,52]
BACE-1	β-secretase that cleaves APP gene in its amyloidogenic pathway.	[45]
α-1-antitripsine	Related with the formation of fibrils of Aβ peptide.	[46]

Aβo: Aβ oligomers; APP: amyloid precursor protein; CSF: cerebrospinal fluid; mRNA: messenger RNA; NFTs: neurofibrillary tangles; PHF: pair helical filaments.

**Table 2:** Electrochemical biosensors for the detection of DNA and RNA AD biomarkers using nanomaterials as electrode modifiers or as labels published over the last ten years.

Nanomaterial	Role	Biomarker	LOD	Linear Range	Selectivity Tested	Real Sample Tested	Ref
CM-GQDs	Electrode modifier	ApoE4 gene	16.7 fM	1.54–30.73 pM	Presence of 100-fold of non-complementary DNA, IgG, cholesterol and glucose oxidase	Human blood plasma	[83]
MB/Fc-GSHs	Electrode modifier	ApoE4 gene	10 fM	10^−8^–10^−14^ M	Non-complementary DNA and one mismatched DNA	Not tested	[84]
GO-CA-PGE	Electrode modifier	miRNA-34a	261.7 nM	0–1.45 µM	Other miRNAs	Not tested	[86]
ERGO/AuNWs	Electrode modifier	miRNA-137	1.7 fM	5.0–750.0 fM	Non-specific oligonucleotides	Human serum with the addition of miRNA	[87]
AuNPs	Electrode modifier	ApoE4 gene	286 nM	10 µM–250 nM	Not tested	Not tested	[89]
Fc-capped AuNPs	Label	ApoE4 gene	0.1 pM	0.1–5 pM	ApoE2/3 with a single base mismatch	DNA extracts from serum samples	[90]

AuNPs: gold nanoparticles; CM-GQDs: curcumin modified graphene quantum dots; ERGO/AuNWs: electrochemically-reduced graphene oxide (ERGO) combined with gold nanowires (AuNWs); Fc-capped AuNPs: ferrocene capped gold nanoparticles modified with streptavidine; GO-CA-PGE: graphene oxide (GO) chemical activated (CA) pencil graphite electrode (PGE); MB/Fc-GSHs: Methylene blue (MB) loaded ferrocenecarboxylic acid (Fc) conjugated graphene @ mesoporous silica hybrids (GSH).

**Table 3:** Electrochemical biosensors for the detection of AD peptide biomarkers using nanomaterials as electrode modifiers or as labels published over the last ten years.

Nanomaterial	Role	Biomarker	LOD	Linear Range	Selectivity Tested	Real Sample Tested	Ref
AuNPs	Electrode modifier	Aβ	22 fM	0.22 pM–2.22 nM	Not tested	Serum samples	[93]
AuNPs	Electrode modifier	Aβ	1.15 pM	2.22–221.6 pM	Not tested	Not tested	[94]
AuNPs	Electrode modifier	p53	0.05 nM	2–50 nM	Not tested	Real plasma samples of MCI and AD patients	[95]
AuNPs	Electrode modifier	Aβ	22.2 pM	111 pM–111 nM	Not tested	Not tested	[96]
AuNPs	Electrode modifier	Aβ	100 fM	100 fM–25 pM.	HSA, IgG and other AD proteins	Serum and plasma samples	[97]
AuNFs AuNPs/Cu-MOFs	Electrode modifier Label	Aβo	0.45 nM	1 nM–2 μM	Aβ 40, 42 monomers and Aβ(40–42)f	Not tested	[98]
AuNPs	Electrode modifier	Aβ	0.57 nM	10 pM–100 nM	BSA	Not tested	[99]
AuNPs-PEDOT	Electrode modifier	Aβo	10^-2^ fM	10^−8^–10^4^ nM	Aβf and monomers	AD mice tissue	[100]
rGO	Electrode modifier	Aβ	2.398 pM	11 pM–55 nM	Aβ40 and ApoE4	Mice and human plasma	[101]
CNT-CuO	Electrode modifier	Aβo	88.6 fM	22 pM–14.6 nM	Not tested	Serum samples	[102]
PS-b-P4VP-CR	Electrode modifier	Aβ	2.21 pM	2.21 pM–221 nM.	Aβ monomers, peptides and complex media with other proteins	Human blood serum	[103]
Au/NiFe_2_O_4_@GO-Ch	Electrode modifier	Aβ	0.66 pM	0.22 pM–222 pM	Aβ 1–40, prostate specific antigens, cortisol and thrombin.	CSF	[104]
Aβ(1–16)-heme-AuNPs	Label	Aβ	10 pM	0.02–1.50 nM	Artificial CSF	Not tested	[105]
AuNPs	Label	Aβ	22.15 fM	0.02–22.2 pM	Brain-derived neurotrophic factor and prostate-specific antigen	Mouse plasma sample	[106]
MB Au@Pt/Au	Electrode modifier Label	p53	66 nM	50–1000 nM.	Not tested	Human plasma samples	[107]
AuNPs	Label	Aβo	100 pM	0.5–30 nM	Aβ40-42 monomers, Aβ(40–42)o, Aβ(40–42)f	CSF	[108]
AuNPs	Label	Aβ	28 pM	0.1–50 nM	Not tested	CSF and rat brain tissues	[109]
Cu^2+^-PEI/AuNPs-hemin	Label	Aβ aggregation	0.2 pM	1 pM–50 nM	Endogenic proteins, Aβ oligomers, metal ions, amino acids and other biological species	CSF of normal and AD mice	[110]
AuNPs	Label	Aβo	45 pM	0.1 nM–0.2 µM	Aβ f and monomers, IgG and thrombin	Blood serum	[111]
AgNPs	Label	Aβo	8 pM	20 pM–100 nM.	Aβf and monomers, IgG, BSA, thrombin and α-synudein	Serum samples	[112]
AuNPs SiO_2_@AgNPs	Electrode modifier Label	Aβo	0.27 pM	1.1 pM–2.2 nM	Aβ40-42 monomers, Aβ(40–42)o, Aβ(40–42)f	Human serum samples	[113]
ZIF-8/Fer	Label	Aβo	10^-5^ μM	10^−5^–10^2^ μM	Aβf and monomers and artificial CSF	Not tested	[114]

Aβ(1–16)-heme-AuNPs: gold nanoparticles conjugated with Aβ(1–16)-heme; Au@Fc-Zn-MOF: gold nanoparticles combined with ferrocene confined in metal-organic framework (Fc-Zn-MOF); AuNFs: gold nanoflowers; Au/NiFe2O4@GO-Ch: gold nanoparticle/nickel ferrite decorated graphene oxide-chitosan nanocomposite; AuNPs: gold nanoparticles; AuNPs/Cu-MOFs: gold nanoparticles combined with copper metal-organic frameworks; AuNPs-PEDOT: AuNPs embedded in a layer of poly(3,4-ethylene dioxythiophene); Au@Pt/Au: core@shell gold platinum nanoparticles; CNT-CuO: carbon nanotubes combined with copper oxide; CNT-PIL: carbon nanotubes combined with polymer ion liquid; Cu^2+^ -PEI/AuNPs-hemin: Polyethyleneimine (PEI) covered AuNPs were used as labels by functionalization of hemin and Cu^2+^; HSA: human serum albumin; MB: magnetic beads; NLC: nanostructure lipid carrier; PS-b-P4VP-CR: nanostructured isoporous poly(styrene-b-4-vinylpiridine) membrane conjugated with congo red; rGO: reduced graphene oxide; SiO_2_@AgNPs: silver nanoparticles and silica nanoparticles; ZIF-8/Fer: Zn zeolite imidazole framework with ferrocene.

**Table 4:** Electrochemical biosensors for the detection of AD protein biomarkers using nanomaterials as electrode modifiers or as labels published over the last ten years.

Nanomaterial	Role	Biomarker	LOD	Linear Range	Selectivity Tested	Real Sample Tested	Ref
FracAu	Electrode modifier	ApoE4	8.78 pM	23.9 pM–293 nM	BSA, human ApoE2 and ApoE3	Not tested	[139]
AuBP@Pt AuPd-PDA	Electrode modifier Label	ApoE4	0.45 pM	1.46 pM–58.6 nM	BSA, human ApoE2 and ApoE3	Not tested	[140]
Au-FLGN	Electrode modifier	Aβ protein	88.6 fM	0.44–284 pM	Hb, heparin, HSA and bilirubin	Serum and CSF	[141]
Microporous gold nanostructure	Electrode modifier	Aβ protein	44.3 fM	0.7 pM–1.6 nM	Hb, heparin, HSA and bilirubin	Plasma and CSF in AD and elderly normal controls	[142]
AuNPs Au@Fc-Zn-MOF	Electrode modifier Label	Aβ protein	6.6 fM	22.2 fM–22.2 nM	HSA, thrombin, BSA, IgG, Aβo, Aβf and monomers	Human serum samples	[143]
3D-Au-PAMAM	Electrode modifier	Tau	0.031 pM	~0.11–91 pM	IgG, Hb, HSA and BSA	Plasma and tissue extract samples	[144]
MWCNTs	Electrode modifier	Aβ protein	44 aM	0.22–22.2 fM	Aβ42, Hb, heparin and bilirubin	Not tested	[145]
MWCNTs@rGO AuNPs	Electrode modifier Label	Tau-441	0.46 fM	0.5–80 fM	Glucose, AA, L-cysteine, HSA	Normal people and MCI patients	[147]
AuNPs	Label	Tau-381	0.42 pM	2.52–25.2 nM	Not tested	Human plasma samples	[148]
Au@rGO AuCuxO@m-CeO_2_	Electrode modifier Label	Aβ protein	7.97 fM	22.2 fM–2.2 nM	Insulin, prostate-specific antigen and alpha fetal protein	Not tested	[152]
MB CdSe@ZnS QDs	Electrode modifier Label	ApoE4	0.37 nM	0.29–5.86 nM	Not tested	Human plasma samples	[154]
MB IrO_2_ NPs	Electrode modifier Label	ApoE4	nM	100–500 nM	Not tested	Ex-vivo AD-induced rat brain samples	[158]
HAP NPs	Label	BACE-1	0.1 U/mL	0.25 to 100 U/mL.	Protein kinase A, alkaline phosphatase, glucose oxidase and alcohol dehydrogenase	Serum samples	[45]
PTCA-CNTs ALP-AAT Ab-Ag NPs	Electrode modifier Label	α-1 antitrypsin	0.01 pM	0.05–20.0 pM	IgG and IgE	Serum samples	[46]

3D-Au-PAMAM: AuNPs-poly(amidoamine) (PAMAM) dendrimer nanocomposite; AA: ascorbic acid; ALP-AAT Ab-Ag NPs: alkaline phosphatase-labeled AAT antibody functionalized silver nanoparticles; AuBP@Pt: gold nanobipyramids coated with Pt; AuCuxO@m-CeO_2_: AuCuxO- embedded mesoporous CeO_2_ Au-FLGN: fern-leaves like gold nanostructured covered with gold; AuNPs: gold nanoparticles; AuPd-PDA: polydopamine nanotubes doped with AuPd; Au@rGO: gold nanoparticles functionalized with reduced graphene oxide; CdSe@ZnS QDs: cadmium-selenide/zinc-sulfide quantum dots; FracAu: fractal gold nanostructures; HAP NPs: hydroxyapatite nanoparticles; Hb: hemoglobin; HSA: human serum albumin; MB: magnetic beads; MWCNTs: multi-walled carbon nanotubes; MWCNTs@rGO: multi-walled carbon nanotubes and reduced graphene oxide; PTCA-CNTs: 3,4,9,10-perylene tetracarboxylic acid/carbon nanotubes.

**Table 5:** Electrochemical biosensors for the detection of neurotransmitters using nanomaterials published over the last ten years.

Nanomaterial	Role	Biomarker	LOD	Linear Range	Selectivity Tested	Real Sample Tested	Ref
SNPs@GO	Electrode modifier	DA	0.2 µM	0.1–100 µM	UA and AA	Not tested	[41]
pGO-GNPs	Electrode modifier	DA	1.28 µM	0.1–30 µM	AA and glucose	Not tested	[162]
Pdop@GR-MWCNTs	Electrode modifier	DA	1 µM	7–297 µM	AA, glucose and different ions	Human serum	[163]
rGS-GNPs	Electrode modifier	DA	0.098 µM	0.1–100 µM	AA	Not tested	[165]
rGS-GNPs	Electrode modifier	NE	200 nM	0.2–10 µM	AA	Not tested	[42]
Fe_2_O_3_ NPs	Electrode modifier	ACh	1.04 µM	2.5–60 µM	Glucose, albumin, DA, NE and AA	Not tested	[167]
Pd/NPG	Electrode modifier	DA	1 µM	1–220 µM	AA, UA, NE, epinephrine and catechol	Not tested	[168]
CHIT/Fe@AuNPs	Electrode modifier	ACh	0.005 µM	0.005–400 µM	AA, UA, DA, lactic acid, heparin sodium, CuSO_4_, KCl, NaCl and MgCl_2_	Healthy and AD plasma samples	[169]
Fe_2_O_3_/rGO-PEDOT	Electrode modifier	ACh	4 nM	4 nM–800 µM	AA, bilirubin, urea, UA, 4-acetamidophenol and glucose	Healthy and AD serum samples	[170]

AA: ascorbic acid; ACh: acetylcholine; CHIT/Fe@AuNPs: nanocomposite of chitosan (CHIT)/gold- coated ferric oxide nanoparticles (Fe@AuNPs); DA: dopamine; Fe_2_O_3_ NPs: magnetic nanoparticles; Fe_2_O_3_/rGO-PEDOT: iron oxide nanoparticles (Fe_2_O_3_ NPs) combined with poly(3,4-ethylenedioxythiophene) (PEDOT)—reduced graphene oxide (rGO) nanocomposite; MWCNTs: multi-walled carbon nanotubes; NE: norepinephrine; Pd/NPG: palladium nanoparticles (Pd) onto a self-supporting nanoporous gold (NPG) wire; Pdop@GR: polydopamine functionalized with graphene; pGO: porous GO; rGS-GNPs: reduced graphene oxide sheets with gold nanoparticles; SNPs@GO: silver nanoparticles covered with graphene oxide; UA: uric acid.

**Table 6:** Electrochemical biosensors for the detection of oxidative stress biomarkers using nanomaterials published over the last ten years and focused on its application for AD detection.

Nanomaterial	Role	Biomarker	LOD	Linear Range	Selectivity Tested	Real Sample Tested	Ref
RGO/AuFe3O4/Pt	Electrode modifier	H_2_O_2_	0.1 µM	0.5 µM–11.5 mM	UA, AA and glucose	Living cells	[172]
ZnO-PVA	Electrode modifier	H_2_O_2_	9.13 nM	1–17 µM	AA, lactic acid, urea and glucose	Human blood serum samples	[173]
CuS/RGO	Electrode modifier	H_2_O_2_	0.27 µM	5–1500 µM	AA, UA, DA, acetaminophen, citric acid and glucose	Human serum and urine samples	[174]
CNPs	Electrode modifier	H_2_O_2_	0.1 pM	0.1 pM–0.1 µM	Glucose, sodium nitrite and UA	Not tested	[175]
PIL/PB/CNT	Electrode modifier	Superoxide anion	0.42 µM	1–228 µM	O_2_, tertbutylhydroperoxide, −OCl, OH, OtBu, ONOO−	Living cells	[176]

AA: ascorbic acid; CNPs: ceria nanoparticles; CuS/RGO: copper sulfide-decorated reduced graphene oxide composites; DA: dopamine; PIL/PB/CNT: ionic liquid polymer (PIL) onto PB nanoparticles (PBNPs) and carbon nanotubes (CNT); RGO/AuFe3O4/Pt: reduced graphene oxide (RGO) nanocomposites decorated with Au, Fe_3_O_4_, and Pt nanoparticles; UA: uric acid; ZnO-PVA: zinc oxide-polyvinyl alcohol nanocomposites.

**Table 7:** Electrochemical biosensors for the multi-detection of AD biomarkers reported during the last ten years.

Nanomaterial	Role	Biomarker	LOD	Linear Range	Selectivity Tested	Real Sample Tested	Ref
MB	Electrode modifier	ApoE4	2.34 pM	2.93–366 pM	Not tested	CSF, serum and plasma	[178]
AuNPs	Label	Aβ	4.21 pM	4.43–2.77 nM			
3D-Au-PAMAM	Electrode modifier	Tau-441	50 fM	0.18–109 pM	BSA, HSA, IgG and Hb	Plasma and tissue samples	[179]
		TDP-43	0.287 pM	1–349 pM			
ABTS-PDDA/CNTs	Electrode modifier	Aβ	0.11 nM	22.2–842 nM	Metal ions, amino acids, AA, DA, UA, lactic acid and glucose	Plasma and hippocampus of normal and AD rats	[181]
		Cu^2+^	0.04 µM	0.1–10 µM			
CNT-PIL NLC	Electrode modifier Label	Aβo	2.2 pM	0.04−8.86 nM	Metal ions, amino acids, other isoforms of Aβ	AD rat brain	[182]
		Aβf	4.4 pM				
Au nanodendrites	Electrode modifier	miRNA-101	91.4 pM	0.1 pM–100 pM	BSA other miRNAs	Serum samples	[183]
		ApoE4	2.09 fM	2.93 fM–2.93 pM			
		Tau	1.29 fM	2.18 fM–2.18 pM			
		Aβ	1.9 fM	22.2 fM–22.2 pM			

3D-Au-PAMAM: AuNPs-poly(amidoamine) (PAMAM) dendrimer nanocomposite; AA: ascorbic acid; ABTS-PDDA/CNTs: 2,2-azinobis-(3-ethylbenzthiazoline-6-sulphonate) (ABTS)- poly(diallyldimethylammonium chloride) (PDDA)/single-walled carbon nanotubes; AuNPs: gold nanoparticles; CNT-PIL: carbon nanotubes combined with polymer ion liquid; DA: dopamine; Hb: hemoglobin; HSA: human serum albumin; MB: magnetic beads; NLC: nanostructure lipid carrier; UA: uric acid.

## Abbreviations

2D	Two-dimensional
3D	Three-dimensional
AA	Ascorbic acid
Aβ	Amyloid β
Aβf	Amyloid β fibrils
Aβo	Amyloid β oligomers
Ach	Acetylcholine
AD	Alzheimer’s disease
ADI	Alzheimer’s disease international
AFM	Atomic force microscopy
AgNPs	Silver nanoparticles
APP	Amyloid precursor protein
AuNPs	Gold nanoparticles
CA	Chronoamperometry
CeNPs	Cerium oxide nanoparticles
CNTs	Carbon nanotubes
CSF	Cerebrospinal fluid
CV	Cyclic voltammetry
DA	Dopamine
DES	Deep eutectic solvents
DNA	Deoxyribonucleic acid
DPV	Differential pulse voltammetry
EDC	1-ethyl-3-(3-dimethylaminopropyl) carbodiimide
ELISA	Enzyme linked immunosorbent assay
FBS	Fetal bovine serum
FISH	Fluorescence in situ hybridization
G8	Group of eight inter-governmental political forum
GCE	Glassy carbon electrode
GDP	Gross domestic product
GO	Graphene oxide
HER	Hydrogen evolution reaction
HRP	Horseradish peroxidase
IrO_2_NPs	Iridium oxide nanoparticles
ITO	Indium tin oxide
LOD	Limit of detection
LSV	Linear sweep voltammetry
MAPT	Microtubule-associated protein Tau
MCH	6-mercapto-1-hexanol
MCI	Mild cognitive impairment
MIPs	Molecular imprinted polymer
miRNAs	microRNAs
MHDA	16-mercaptohexadecanoic acid
MOFs	Metal-organic frameworks
MRI	Magnetic resonance imaging
mRNA	Messenger RNA
MWCNTs	Multi-walled carbon nanotubes
NE	Norepinephrine
NFTs	Neurofibrillary tangles
NiFe_2_O_4_	Nickel ferrites
NIR	Near infrared
NKB	Neurokinin B
NHS	N-hydroxysuccinimide
OECT	Organic electrochemical transistors
PBS	Phosphate buffer saline
PCR	Polymerase chain reaction
PEI	Polyethyleneimine
PET	Positron emission tomography
PHFs	Pair helical filaments
POC	Point-of-care
PrPc	Cellular prion protein
QDs	Quantum dots
rGO	Reduced graphene oxide
RNA	Ribonucleic acid
ROS	Reactive oxygen species
RSD	Relative standard deviation
SAM	Self-assembled monolayer
SELEX	Selective evolution of ligands by exponential enrichment
SEM	Scanning electron microscopy
SPCEs	Screen-printed carbon electrodes
ssDNA	Single-stranded DNA
SWCNTs	Single-walled carbon nanotubes
SWV	Square wave voltammetry
TDP-43	TAR DNA-binding protein 43
UA	Uric acid
WHO	World health organization
WOR	Water oxidation reaction
